# Case study on communicating with research ethics committees about minimizing risk through software: an application for record linkage in secondary data analysis

**DOI:** 10.1093/jamiaopen/ooae010

**Published:** 2024-02-29

**Authors:** Cason Schmit, Alva O Ferdinand, Theodoros Giannouchos, Hye-Chung Kum

**Affiliations:** Population Informatics Lab, Texas A&M University, College Station, TX 77843, United States; Department of Health Policy & Management, Texas A&M University, College Station, TX 77843, United States; Department of Health Policy & Management, Texas A&M University, College Station, TX 77843, United States; Population Informatics Lab, Texas A&M University, College Station, TX 77843, United States; Department of Health Policy & Organization, The University of Alabama at Birmingham, School of Public Health, Birmingham, AL 35233, United States; Population Informatics Lab, Texas A&M University, College Station, TX 77843, United States; Department of Health Policy & Management, Texas A&M University, College Station, TX 77843, United States; Department of Computer Science and Engineering, Texas A&M University, College Station, TX 77843, United States

**Keywords:** Ethics Committees, Research, Privacy, nominal group technique, Delphi Technique, Medical Record Linkage

## Abstract

**Objective:**

In retrospective secondary data analysis studies, researchers often seek waiver of consent from institutional Review Boards (IRB) and minimize risk by utilizing complex software. Yet, little is known about the perspectives of IRB experts on these approaches. To facilitate effective communication about risk mitigation strategies using software, we conducted two studies with IRB experts to co-create appropriate language when describing a software to IRBs.

**Materials and Methods:**

We conducted structured focus groups with IRB experts to solicit ideas on questions regarding benefits, risks, and informational needs. Based on these results, we developed a template IRB application and template responses for a generic study using privacy-enhancing software. We then conducted a three-round Delphi study to refine the template IRB application and the template responses based on expert panel feedback. To facilitate participants’ deliberation, we shared the revisions and a summary of participants’ feedback during each Delphi round.

**Results:**

11 experts in two focus groups generated 13 ideas on risks, benefits, and informational needs. 17 experts participated in the Delphi study with 13 completing all rounds. Most agreed that privacy-enhancing software will minimize risk, but regardless all secondary data studies have an inherent risk of unexpected disclosures. The majority (84.6%) noted that subjects in retrospective secondary data studies experience no greater risks than the risks experienced in ordinary life in the modern digital society. Hence, all retrospective data-only studies with no contact with subjects would be minimal risk studies.

**Conclusion:**

First, we found fundamental disagreements in how some IRB experts view risks in secondary data research. Such disagreements are consequential because they can affect determination outcomes and might suggest IRBs at different institutions might come to different conclusions regarding similar study protocols. Second, the highest ranked risks and benefits of privacy-enhancing software in our study were societal rather than individual. The highest ranked benefits were facilitating more research and promoting responsible data governance practices. The highest ranked risks were risk of invalid results from systematic user error or erroneous algorithms. These societal considerations are typically more characteristic of public health ethics as opposed to the bioethical approach of research ethics, possibly reflecting the difficulty applying a bioethical approach (eg, informed consent) in secondary data studies. Finally, the development of privacy-enhancing technology for secondary data research depends on effective communication and collaboration between the privacy experts and technology developers. Privacy is a complex issue that requires a holistic approach that is best addressed through privacy-by-design principles. Privacy expert participation is important yet often neglected in this design process. This study suggests best practice strategies for engaging the privacy community through co-developing companion documents for software through participatory design to facilitate transparency and communication. In this case study, the final template IRB application and responses we released with the open-source software can be easily adapted by researchers to better communicate with their IRB when using the software. This can help increase responsible data governance practices when many software developers are not research ethics experts.

## Introduction

### Big data ethics, software, and IRBs

Increasingly, researchers are leveraging various health information datasets (eg, claims data, electronic health records, discharge data) to advance medical care and generalizable knowledge. While independent datasets are informative, linking individual-level records across disparate datasets is critical to further investigations.^1^^,^^2^ The process of linking individual-level records is referred to as “Record Linkage” (RL) or “patient matching” and has historically involved the disclosure of all patient identifiers contained in the converging datasets.^3–6^ These identifiers are needed to determine which records from each dataset should be combined. However, fully disclosing identifiers for RL challenges patient privacy and trust in the research enterprise.^6–8^

Institutional Review Boards (IRBs) are charged with protecting human research subjects, including their identifiable data; however, traditional RL challenges this oversight because the enhanced benefits of linking existing datasets are accompanied by new disclosure risks and new regulatory and ethical considerations.^9^^,^^10^ For example, RL implicates laws protecting identifiable data (ie, Health Insurance Portability and Accountability Act of 1996 and the Common Rule).^11–14^ Moreover, researchers have noted limitations of many existing data governance systems given the requirements for cyberinfrastructure to manage sensitive data.^15–17^

In response, several privacy-enhancing RL protocols provide alternatives to limit disclosure of patient identifiers.^6^^,^^8^^,^^18–24^ These approaches often rely on complex software to mitigate risks. However, little is known about the perspectives of research ethics experts—who routinely serve on IRBs and research ethics committees—on privacy-enhancing RL protocols. Research ethics perspectives are critical to guide unfolding advances in human subject research.

For researchers, understanding research ethics perspectives on privacy-preserving techniques has practical importance given that effective communication with IRB professionals can result in timely approval of research plans. Since informed consent is rare in retrospective secondary data research, IRBs must carefully consider research risks and benefits on behalf of the data subjects.^25^ This includes considerations of highly technical research protections like secure computer systems and software used to protect privacy. Yet, communicating about the relevant technical details of a software to minimize risk is not trivial. Misunderstandings between researchers and IRB reviewers on these technical protections and protocols can result in extended and unnecessary delays in research approvals. In theory, use of a privacy-enhancing protocol during RL should reduce the associated risks of a study requiring RL, but IRBs must understand the risk-mitigating effect of the privacy-enhancing protocol to make the appropriate evaluations of risks and benefits.

This paper seeks to understand research ethics experts’ perspectives and how to best communicate with them about the use of incremental disclosure of identifying information during the RL process, implemented in an open-source software called *Minimum Necessary Disclosure for Interactive RL (MiNDFIRL)*.^26^^,^^27^ To this end, we conducted two studies with participants from the research ethics community serving on IRB boards. This research informed the development of the MiNDFIRL software and will be useful to researchers, clinicians, and research ethics personnel interested in maximizing the utility of RL in secondary data studies while simultaneously supporting the privacy interests of human subjects. This research is the first case study—to our knowledge—that engages the research ethics community in the participatory design of software to enhance privacy protection in secondary data research.

### MiNDFIRL: record linkage prototype software

**Figure 1.**
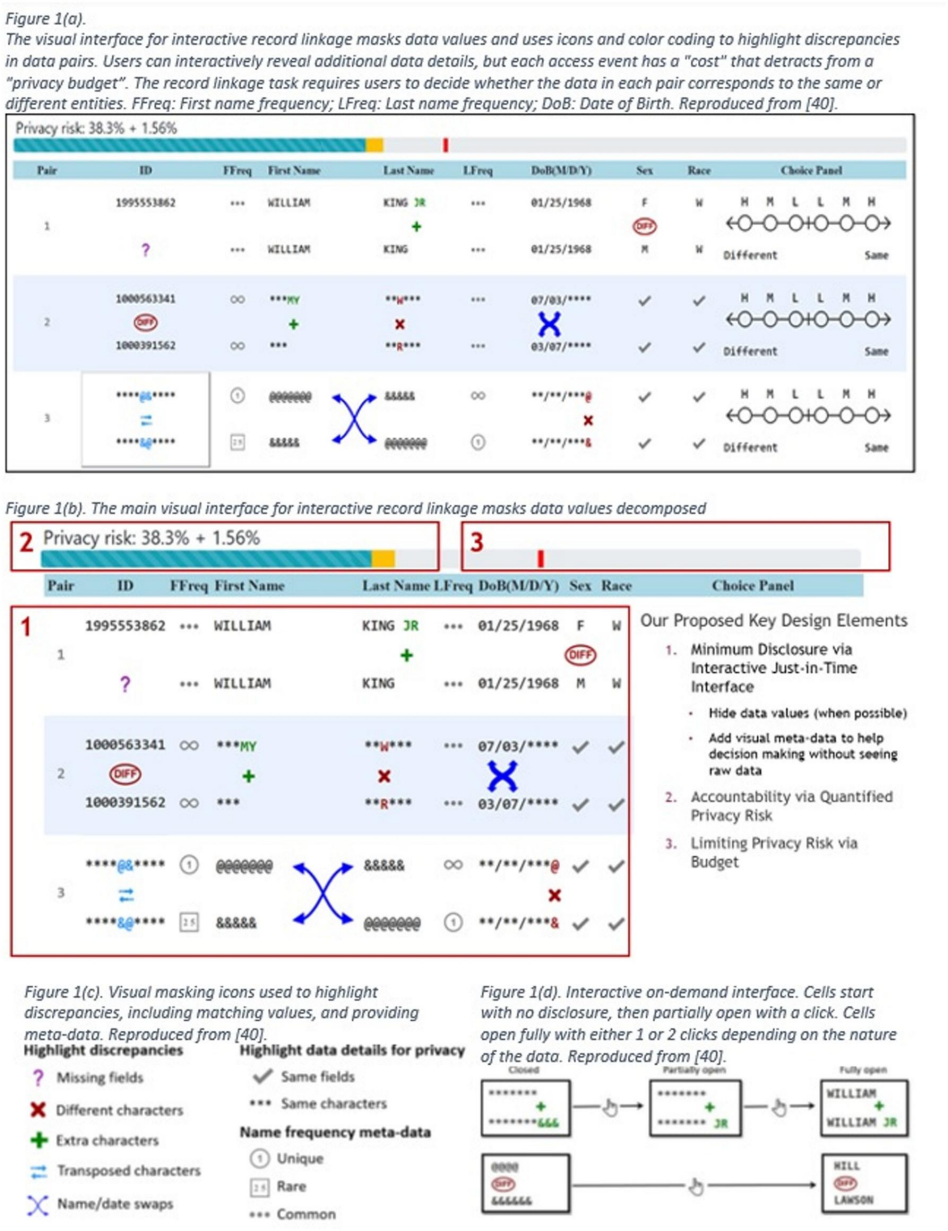
MiNDFIRL prototype software.

**Figure 2.**
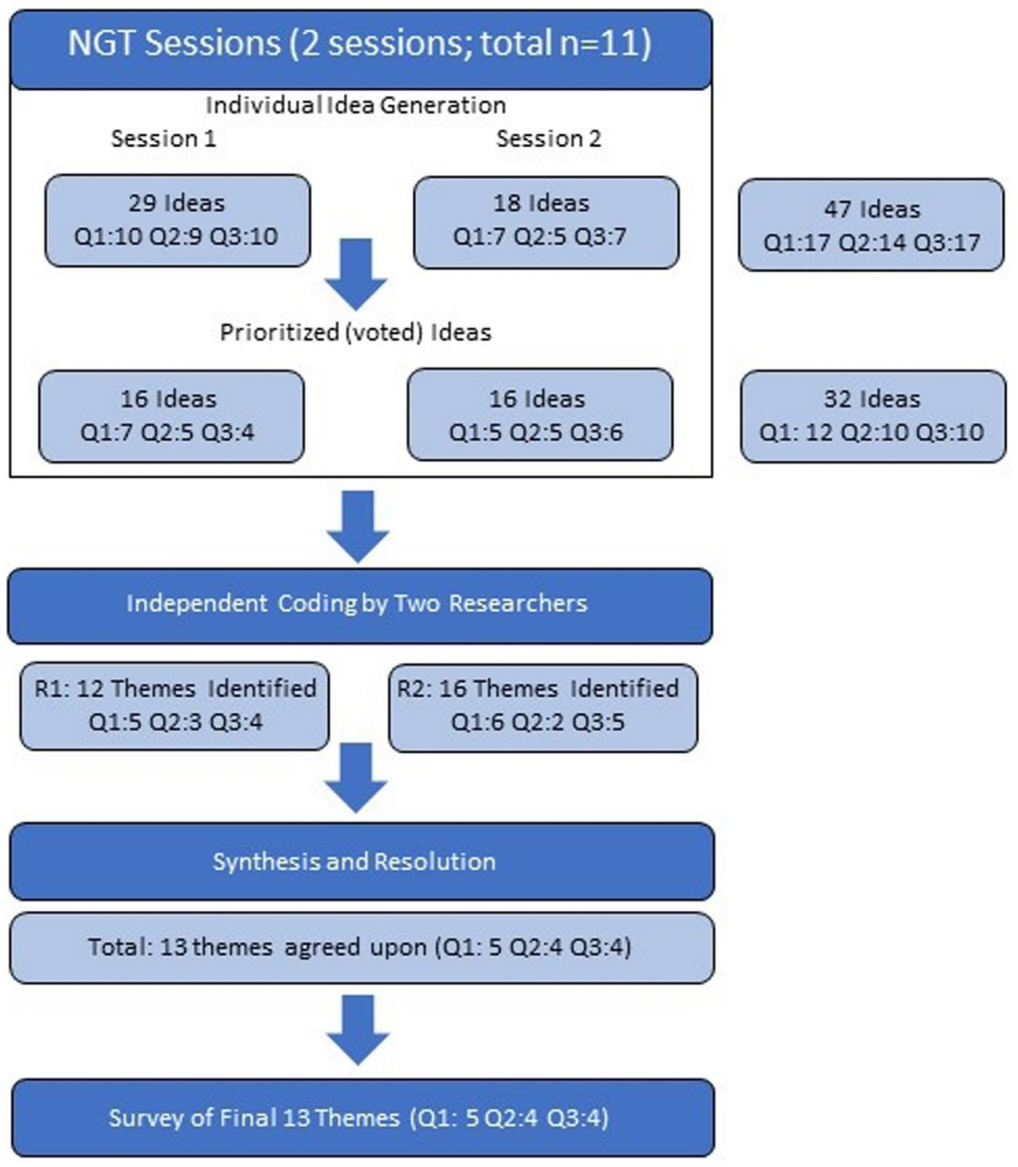
NGT process of idea generation and consensus building.

MiNDFIRL is an open-source prototype software that provides privacy protection through controlled and transparent disclosure of personally identifiable information. It uses a hybrid human-computer RL system: Automated machine learning models^26^ efficiently match easy records, and privacy-enhanced manual resolution is used for complicated records.^23^^,^^24^ This hybrid model can achieve high-quality RL. Three key design elements assist privacy conscious decision making in manual RL (Figure 1A and B): (1) interactive just-in-time minimum disclosure (Figure 1D), (2) accountability via quantified privacy risk, and (3) limiting privacy risk via budget.^23^ This approach also utilizes coding and separation of data, the addition of fake data (ie, obfuscation via chaffing), and minimum disclosure via recoding (Figure 1C).^6^ Together these features effectively implement the “minimum necessary” ethical principle for privacy protection through the privacy-by-design approach. Controlled experiments demonstrated that MiNDFIRL can enhance privacy while supporting legitimate access for human decision making.^23^^,^^24^ The experiments also suggest that quality can be diminished when privacy standards are too stringent.^23^^,^^24^ While these technical approaches benefit data subjects, they add additional layers of complexity for research ethics committees to understand.

As part of the participatory design of MiNDFIRL, we engaged different stakeholder groups to create companion resources: (1) a template dynamic online privacy statement, (2) an IRB application and response template, and (3) a template Data Use Agreement. These resources are freely available alongside the MiNDFIRL software on GitHub^28^ to help users effectively communicate with appropriate stakeholders.

### Objectives

Here, we describe the process of engaging with research ethics experts to develop the template IRB application and template responses. The primary objective was to co-create a companion document to the software, to facilitate communication with IRBs about conducting secondary data studies using the MiNDFIRL software.

## Materials and methods

### Design

We conducted two studies utilizing nominal group technique (NGT) and Delphi methods with participants that were research ethics experts. First, NGT was used to identify the most critical issues that a principal investigator (PI) should address when communicating with IRB members about the benefits and risks of a secondary data-only study involving RL and privacy-enhancing technology.^29^^,^^30^ Building on the NGT results, we developed the initial draft of the IRB template and PI responses for a generic study using the MiNDFIRL software. We then used the Delphi technique—useful for developing communication strategies where divergent viewpoints exist—to iteratively improve the documents with confidential feedback from a panel of research ethics experts.^29^^,^^31^ The study received ethical approval by the IRB of Texas A&M University.

### NGT sessions

We conducted two NGT sessions. We conducted the first session at the Advancing Ethical Research conference and recruited from conference attendees.^31^^,^^32^ We facilitated a second online session after the conference recruiting from professional mailing lists and by consulting IRB websites. In person, participants were each given a $25 gift card and also entered a raffle to receive an additional $100 gift card. Online participants were each given a $20 gift card and entered a raffle for an additional $50.

The study team drafted three questions related to the potential benefits, risks, and information required by the IRB community as follows,

1. What do you perceive as the benefits of using the MiNDFIRL approach for database RL?
2. What do you perceive as the risks for subjects of data when using the MiNDFIRL approach for database RL?
3. For research using the MiNDFIRL approach for RL, what other information would you need to know if you were serving on the IRB as the public representative for reviewing and approving an IRB application?

To promote better understanding and real-world idea generation for our framework, we provided a 15-min online tutorial.^33^ The tutorial presented the linkage framework and gave participants hands-on experience using MiNDFIRL to link records across two databases. Afterward, the NGT sessions followed the typical three-phase structure (Figure 2) which lasted 45-60 min. First, we gave participants a total of 30 min (10 min per question) to individually build a list of responses to each question. Next, all participant responses were gathered, shared, and clarified in open group discussions for each question. Common responses were combined by participants into broader themes. Finally, participants were asked to vote on the two most important themes per question. Four researchers independently conducted thematic analyses on the results to create a single list of themes across both groups. Participants in both groups were emailed this combined list to prioritize and rank-order two of the final responses based on perceived level of importance (primary and secondary).

### Delphi study process

**Figure 3.**
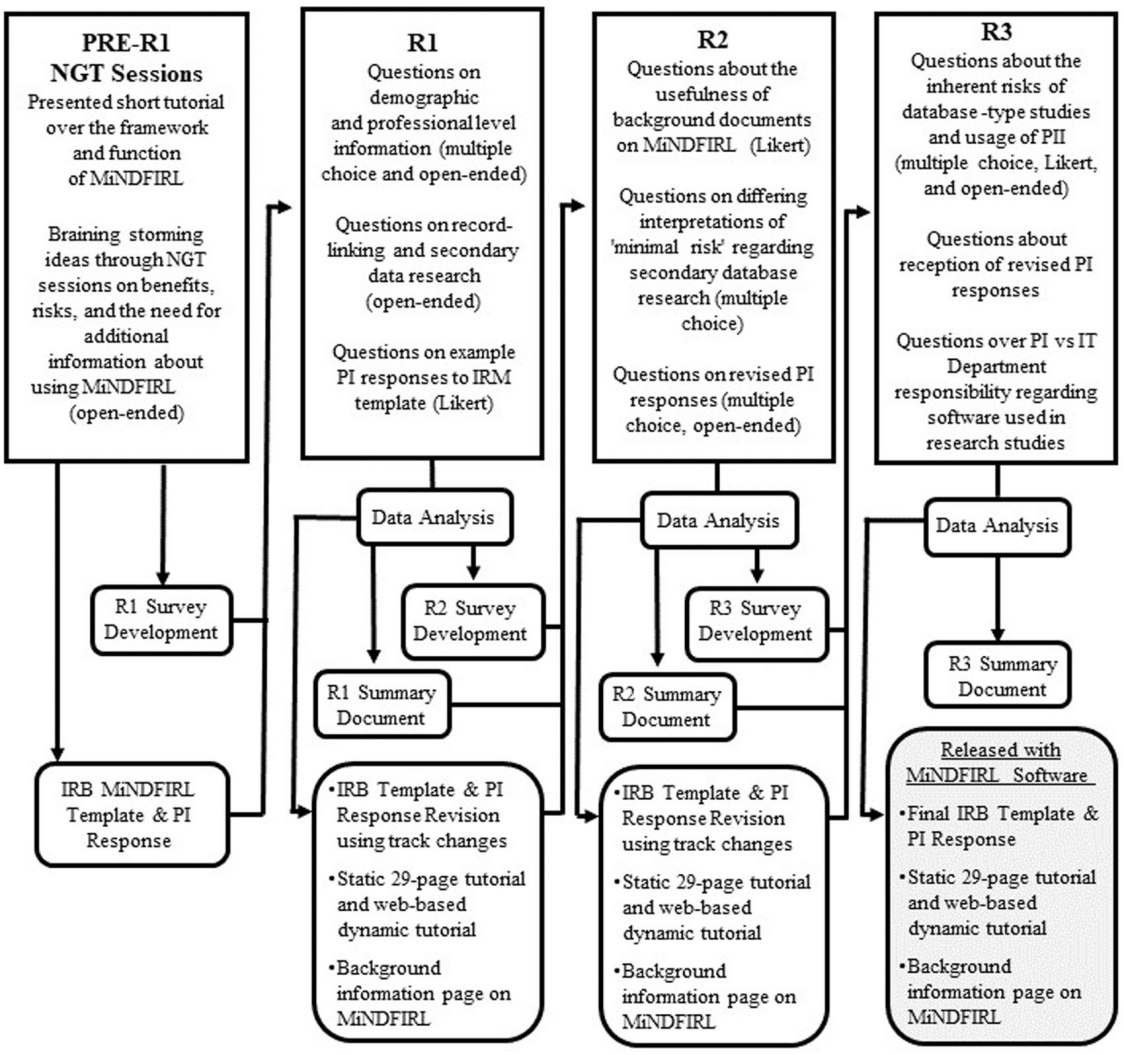
Delphi process.

Building on the NGT results, we developed and refined a template IRB application and template responses through a 3-rouund Delphi study. We recruited experts from professional mailing lists and by consulting IRB websites. During the first round, we collected demographic and professional information on participants (Figure 3). We also included questions on RL and secondary data research to assess familiarity with the topic. Each Delphi Round contained a mix of open-ended questions and 5-point Likert scale questions related to the IRB template.

In each round, participants were asked to provide feedback relating to the content of the IRB template form and the draft PI responses, including whether the sections provided required information for the approval of future studies that will use the MiNDFIRL software. After the first and second round, we revised both template documents to incorporate participant comments and respond to identified issues. The research team discussed participants’ comments and all proposed revisions after completion of each Delphi round. In Rounds 2 and 3, we also provided participants with a summary of the participant feedback from the previous round and “tracked-changes” (eg, “redline”) version of the revised IRB template to enable them to see the specific changes that were made based on their feedback. Whenever participant feedback suggested a divergence of opinions or suggestions, we devised questions to explore the divergence by explicitly raising the issues and providing participants with the opportunity to provide additional feedback. For example, in Round 2 participant feedback suggested differing interpretations of “minimal risk” regarding secondary data research. Consequently, we included focused questions in Round 3 that would allow for better understanding of the diverse perspectives. In response to Round 1 feedback requesting information on the software, we provided additional documents about MiNDFIRL to participants to familiarize themselves with the software in Round 2.

Although the Delphi technique does not demand a specific threshold for consensus, our consensus criteria cutoff was negative feedback by three or fewer individuals, which was conservative and higher than previous Delphi studies.^29^^,^^31^^,^^34^ Participants were given just over a week to complete each round of the survey with two reminder emails sent out within that time to reduce attrition. We compensated participants gradually for each round amounting to $100 for those who completed all three rounds.

## Results

### Study 1 NGT results

In total, 11 individuals participated across both NGT sessions. Their professional affiliation included direct IRB-related roles, such as program director, IRB board member, and research ethics research positions. All participants had appointments in academic, governmental, or health system settings. After discussion and clarification, participants in both groups generated a total of 34 ideas for all three questions. Importantly, there was general consensus about most ideas and issues raised, as well as similarities and overlaps in many of the responses, indicating saturation. After de-duplications across the two groups, 13 ideas remained (Figure 2).

**Figure 4.**
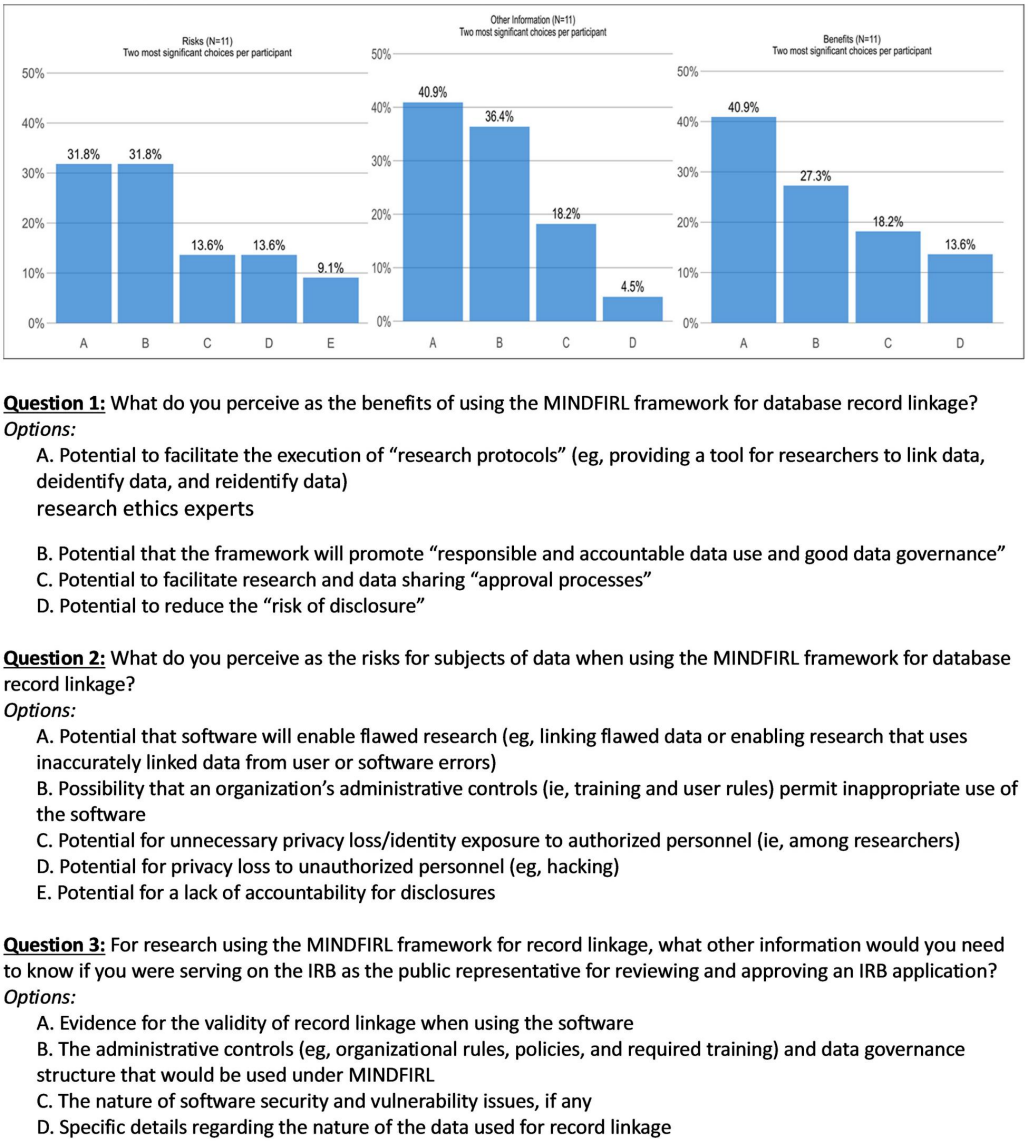
Emerging themes from research ethics experts from NGT sessions.

Figure 4 depicts the final ranked ideas from both sessions. Participants ranked the most important MiNDFIRL benefits as the potential to facilitate the execution of research protocols (eg, providing a tool for researchers to link, de-identify, and re-identify data as appropriate) and the potential that the software will promote responsible and accountable data use and good data governance. For the potential risks, participants ranked the potential that the software will enable flawed research (eg, linking flawed data or enabling research that uses inaccurately linked data from user or software errors) and the possibility that an organization’s administrative controls inadequately dissuade inappropriate use of the software as the most profound risks. The highest ranked ideas for additional information were the validity of RL when using the software and the administrative controls and data governance. Overall, participants indicated that the hands-on online tutorial of the software prior to the NGT session was valuable in raising their awareness of the RL process and helped them generate informed ideas in the sessions.

### Study 2 Delphi results

Of the 18 research ethics experts, 17 fully completed round 1, 15 round 2, and 13 round 3, with an overall response rate of 72.2%. The mean age of all participants was 45.1 years (SD =10.2), 88.2% were females, with at least masters (47.1%) or bachelor’s (29.4%) level education. The majority were certified IRB professionals (82.4%) for approximately 6 years. In terms of professional roles, all were IRB staff and 70.6% were also IRB board members.

The principal Delphi results comprised the final template IRB application and the template responses developed through the three-round Delphi process. We included these template documents in the supplementary material. Below we describe the results from each Delphi round in detail.

The round 1 responses (Table 1) indicated that participants were familiar with the process of RL, secondary data research, and the new Common Rule provisions relating to secondary data studies, while 47.1% were also aware of privacy-enhancing RL methods. Participants indicated that a potential confidentiality breach was the prevailing risk in secondary data-only studies using RL (100%), followed by possible privacy invasion for the subjects or their family (64.7%), and by psychological risks (35.3%).

**Table 1.** Round 1—demographics, professional level information, and MiNDFIRL responses (*N* = 17)

Characteristics	Total/mean	Percent/SD
Demographics		
**Gender, female [*N*, %]**	15	88.2
**Age [mean, SD]**	45.1	10.5
**Educational level [*N*, %]**		
Some college credit, no degree	17	100.0
Bachelor’s degree	2	11.8
Master’s degree	5	29.4
Doctoral degree	8	47.1
Professional level information	2	11.8
**CIP Certified [*N*, %]**	14	82.4
**Year CIP Certified [mean, SD]**	6.4	5.6
**Professional Role [*N*, %]**		
Professional role: IRB Staff	17	100.0
Professional role: IRB Board Member	12	70.6
Professional role: Other (eg, Ethicist, director of HSR)	3	17.6

**Questions on RL and secondary data research**	N	%

**What is a secondary data research study?**		
Secondary data research study: use of existing data	17	100.0
NOT Secondary data research study: interviews with groups of people	16	94.1
Secondary data research study: no researcher interaction with data subjects	16	94.1
Secondary data research study: collected for other purposes (including other research questions), but used to answer this research question	16	94.1
**Familiar with new Common Rule**	16	94.1
**Familiar with any privacy preserving record linkage methods**	8	47.1
In your opinion, what are potential risks involved in a retrospective database only study involving PII for record linkage? From the list below, please select ALL the potential risks in such a study.		
Potential breach of confidentiality	17	100.0
Possible invasion of privacy of subject or subject’s family	11	64.7
Social/economic risks	9	52.9
Psychological risks	6	35.3
Physical risks	1	5.9

**Questions about responses on MiNDFIRL in Round 1**	N	%

**As an IRB reviewer, how satisfied are you that the provided response to this question addresses research issues associated with data protection and confidentiality in the record linkage process?**	17	100.0
Completely satisfied	7	41.2
Somewhat satisfied	6	35.3
Neutral (neither satisfied or dissatisfied)	1	5.9
Partially dissatisfied	3	17.6
Completely dissatisfied	0	0.0
**Are there essential administrative controls that should be applied for this research to be approved?**		
Yes	12	70.6
**As an IRB reviewer, do you have any fundamental concerns about the description of administrative controls in this document?**		
No, I do not have any fundamental concerns	16	94.1
**How confident are you that the investigators' precautions and monitoring plans to avoid or minimize risks are sufficient and appropriate?**	17	100.0
Extremely confident	6	35.3
Confident	4	23.5
Moderately confident	7	41.2
Minimally confident	0	0.0
Not at all confident	0	0.0
**In your judgment, how important is it for the IRB application to obtain additional information on the storage of linked data for future use?**	17	100.0
Extremely important	14	82.4
Important	3	17.6
Moderately important	0	0.0
Minimally important	0	0.0
Not at all important	0	0.0
**If you were an IRB reviewer, does the provided IRB application form request all of the information you would need to make a determination on human subject research in the database only studies involving record linkage?**		
Yes	14	82.4
**What information about future uses would you need to know in order to approve the current research? (Open ended question thematic content analysis)**	17	
Level of identifiability (identifiable, coded, deidentified) of data being kept for future use	7	41.2
Maintenance, storage, and sharing (eg, for how long; how will it be transmitted; name of responsible institution for operation; process for tracking disposition of data)	6	35.3
Purpose of future uses (eg, data linkage)	5	29.4
Disclosure of variables (including Identifiers)	4	23.5
Who will get access	3	17.6
Restrictions for future use	3	17.6
Process for getting permission including agreements and requirements	3	17.6
No re-identification or future contact with subject	2	11.8
Level of consent given (eg, unspecified future use or only for specific uses)	1	5.9
Process for subject withdrawal from repository	1	5.9
Results of repository research shared with subjects	1	5.9

During the first round, all 17 participants revealed that they did not have any fundamental concerns about the procedures of MiNDFIRL to conduct RL described in the IRB template. 76.5% were completely or somewhat satisfied with the data protection provisions, and 70.6% deemed the template language on administrative controls as essential to approve future research. Almost all participants did not have any concerns about the description of administrative controls in the document. In terms of the investigators’ precautions and monitoring plans to minimize risks, all respondents were extremely confident (35.3%), confident (23.5%), or moderately confident (41.2%) that the description was sufficient. In total, 82.4% of participants indicated that the template IRB application provides sufficient information for determination on human subject research in the secondary data-only studies involving RL for IRB reviewers. Thematic analysis of the open-ended question ‘What other information is needed about future uses to approve the current project’, identified the top three themes as (1) whether identifiers would be stored for future use (41.2%), (2) how the data would be maintained, used, and shared (35.3%), and (3) for what purposes would the data be used (29.4%). Major suggestions included revising the language of some IRB questions, additional information about MiNDFIRL, and proposed PI responses.

Based on this feedback, we drafted two more documents about MiNDFIRL that could be submitted with the IRB application. One was a 29-page static tutorial that included a link to a web-based dynamic tutorial and the other was a three-page background information about the software (See appendix). In round 2 (Table 2), all respondents found both documents very useful (66.7% vs 26.7%), useful (26.7% vs 33.3%), or somewhat useful (6.7% vs 40.0%) for understanding MiNDFIRL. These responses seem to indicate that on average participants found the much longer tutorial more useful compared to the short background summary. All the revisions were widely accepted by participants, and minimum 12 out of the 15 participants in this round indicated that the revisions and language changes throughout the different parts of template improved the document. Minor revisions were suggested in the parts related to the description of the overall aspects of the protocol and the ways to protect privacy interests of participants.

**Table 2.** Round 2—MiNDFIRL responses (*N* = 15).

Questions about responses on MiNDFIRL in Round 2	N	%
**Did you find the attachment containing a tutorial and training for MINDFRL useful?**	15	100.0
Very useful	10	66.7
Useful	4	26.7
Somewhat useful	1	6.7
Slightly useful	0	0.0
Not at all useful	0	0.0
**How useful was the tutorial/training attachment on MINDFRL for your understanding of the software?**	15	100.0
Very useful	4	26.7
Useful	5	33.3
Somewhat useful	6	40.0
Slightly useful	0	0.0
Not at all useful	0	0.0
**Is the revised response regarding laws the data are subject to, and rules/policies about the storage and transmission of data better than the previous PI response?**		
Yes	14	93.3
**Is the revised response about PII needed for the study and how MINDFRL will be used to enhance privacy better than the previous PI response?**		
Yes	12	80.0
**Is the revised response about special concerns regarding information in the dataset (such as HIV status) in regard to eventually linked data better than the previous PI response?**		
Yes	14	93.3
**Is the revised response regarding how MINDFRL minimizes the risk of unauthorized disclosures related to accessing PII during record linkage better than the previous PI response?**		
Yes	15	100.0
**Is the revised response about the usage and storage of PII within MINDFRL better than the previous PI response?**		
Yes	14	93.3
**Is the revised response about specific administrative controls at your organization better than the previous response?**		
Yes	12	80.0

In the final round (Table 3), 46.2% were extremely satisfied with the revised IRB application template and the rest were somewhat satisfied. In general, participants thought the summary of previous round was good and facilitated more thinking on these topics as indicated by one participant who stated that “The feedback was thorough and made me think about things I hadn't considered when I completed the survey.” 69.2% strongly agreed or agreed with the statement “use of the MiNDFIRL software will further reduce risk to the minimum necessary to conduct reliable RL” and the remaining four were neutral. All experts unanimously reported that IRB applications that describe using specialized software need to report whether the IT department reviewed and approved use.

**Table 3.** Round 3—MiNDFIRL responses (*N* = 13).

Questions about MiNDFIRL in Round 3	N	%
**How satisfied are you with this version of the IRB application template overall?**	6	46.2
Extremely satisfied:	7	53.8
Somewhat satisfied:	0	0.0
Neutral (neither satisfied or dissatisfied)	0	0.0
Somewhat dissatisfied	0	0.0
Extremely dissatisfied	0	0.0
**Do you agree with the statement “the use of the MINDFIRL software will further reduce risk to the minimum necessary to conduct reliable record linkage”?**	13	
Strongly agree	2	15.4
Agree	7	53.8
Neutral	4	30.8
Disagree	0	0.0
Strongly disagree	0	0.0
**Within an IRB application, is it important to ask “Has the IT department of your organization reviewed and approved the use of MINDFIRL?”**	13	100.0
Very important	6	46.2
Moderately important	3	23.1
Important	4	30.8
Slightly important	0	0.0
Not important	0	0.0
**How important do you think it is to insert the question “Which IT department is managing the server that has MINDFIRL installed and will store the PII?” into the IRB template?”**		
Very important	5	38.5
Moderately important	0	0.0
Important	5	38.5
Slightly important	1	7.7
Not important	2	15.4
**How important do you think it is to insert the question “Who maintains the MINDFIRL access logs? Who has access to the logs? Who will review the logs? How often?” into the IRB template?**		
Very important	5	38.5
Moderately important	2	15.4
Important	5	38.5
Slightly important	0	0.0
Not important	1	7.7
**Do you agree with the following statement, “Despite the use of the MINDFIRL software, which aims to reduce unnecessary disclosures for purposes of record linkage, all database studies have an inherent risk of unexpected disclosures due to a potential breach of the computer system hosting the data?”**		
Strongly agree	8	61.5
Agree	5	38.5
Neutral	0	0.0
Disagree	0	0.0
Strongly disagree	0	0.0

Based on comments from the previous round, we also asked multiple questions about the risk determination of secondary data-only studies using PII. We provide additional details in Table 4. 76.9% strongly agreed or agreed on the statement that inherent risks of unexpected disclosures due to a potential breach of the computer system are no more than those encountered in ordinary life in the modern digital society considering the numerous breaches of computer systems in the news periodically. Most agreed or strongly agreed (84.6%) that secondary data-only studies using regular health data, excluding highly confidential data (eg, HIV, mental health, substance abuse data) with access to PII did not pose greater than minimal risk. The other 15.4% were neutral. Opinions on the level of minimal risk diverged for sensitive data.

**Table 4.** Round 3—risk determination (*N* = 13)

Questions about secondary data-only studies and risk level	*N*	%
**Do you think there are database-only studies (ie, there is no contact with participants) that may constitute greater than minimal risk?**		
Yes	10	76.9
**Do you agree with the following statement, “Risks experienced in ordinary life in the modern digital society includes risk of privacy violation and loss of confidentiality due to linked data because data is ubiquitous and different datasets are linked for various purposes?”**		
Strongly agree	3	23.1
Agree	9	69.2
Neutral	0	0.0
Disagree	1	7.7
Strongly disagree	0	0.0
**Do you agree with the following statement, “The nature of the risk in a database only study is not substantively different than risks experienced in ordinary life?”**		
Strongly agree	2	15.4
Agree	9	69.2
Neutral	0	0.0
Disagree	2	15.4
Strongly disagree	0	0.0
Do you agree with the following statement? “The inherent risk of unexpected disclosures due to a potential breach of the computer system hosting the data is **no more than those encountered in ordinary life** in the modern digital society considering the numerous breaches of computer systems in the news periodically”.		
Strongly agree	2	15.4
Agree	8	61.5
Neutral	3	23.1
Disagree	0	0.0
Strongly disagree	0	0.0
Do you agree with the following statement? “As such, a database only research study involving data using PII is a minimal risk study as it poses **no greater risks than those ordinarily encountered in daily life**”.		
Strongly agree	2	15.4
Agree	6	46.2
Neutral	2	15.4
Disagree	2	15.4
Strongly disagree	1	7.7
Overall, do you believe that the risks of a database-only study (with access to PII) **are greater than minimal risk** for “regular health data” (ie, excluding highly confidential data such as HIV, mental health, substance abuse data)?		
Strongly agree	0	0.0
Agree	0	0.0
Neutral	2	15.4
Disagree	10	76.9
Strongly disagree	1	7.7
Do you agree with the following statement “Overall, do you believe that the risks of a database-only study (with access to PII) **are greater than minimal risk** for sensitive data such as HIV, mental health, substance abuse data?”		
Strongly agree	1	9.1
Agree	5	45.5
Neutral	3	27.3
Disagree	2	18.2
Strongly disagree	0	0.0

## Discussion

In these studies, we obtained robust input on using MiNDFIRL to reduce risk in secondary data-only studies from professionals who routinely consider issues about ethical scientific research.

### General observations

First, we observed that the highest ranked risks and benefits in our NGT sessions were societal rather than individual (eg, unnecessary privacy loss, individual harm). Our NGT findings showed that research ethics professionals consider privacy-enhancing technology as having significant societal benefits by facilitating more research and promoting responsible data governance practices. Similarly, the highest ranked risks by our research ethics panel were those that affect society (eg, risk of invalid results from systematic user error or erroneous RL algorithms).

This focus on societal (rather than participant) risks and benefits is significant. Research ethics are predominately participant-focused for good reason. A history of misconduct impelled foundational cannons of research ethics—like the Belmont Report—to prioritize the benefits and risks affecting the research participants (eg, respect for persons, autonomy, informed consent).^34^ Consequently, societal issues of common good are often secondary considerations. These societal considerations are typically more characteristic of public health ethics as opposed to the bioethical approach of research ethics.^35^ Consistent with these findings, we note that the ethical issues raised in most secondary data studies align better with public health ethics—which places greater weight on societal risks and benefits, such as promoting the common good—than the bioethical approach^36–38^—which relies heavily on informed consent.

### Implications on the use of software like MiNDFIRL

Overall, participants noted that the benefits outweigh the risks when using the MiNDFIRL software. Participants noted the inadequacies of traditional RL approaches that lack accountability and transparency. Privacy-enhancing software, like MiNDFIRL, has the potential to facilitate the approval of research protocols, improve transparency, and support more robust data governance in research involving RL.

However, this potential is largely dependent on a few factors. First, researchers must be able to adequately communicate issues about privacy-enhancing software like MiNDFiRL to their IRBs. Review processes might be unnecessarily delayed if IRBs are unfamiliar with complex software used in research. Our study directly addresses this need by providing researchers with insight on the range of questions IRB members might have and the types of responses that can facilitate understanding and address concerns. Second, the benefits of privacy-enhancing software, like MiNDFIRL, are likely also dependent on institutions’ administrative controls (eg, training, organizational rules, policies) for the responsible use of privacy-enhancing technology. Our findings can inform organizational and policy-specific parameters that are relevant to the ethical use of privacy-enhancing RL software in research (eg, data security, training, disclosure and sharing rules, error management).

### Differences in ethical research review in IRB participants

While all Delphi panel participants reported satisfaction with the developed template language, this language will not be perfect for all IRB reviewers. Our findings show fundamental disagreements in how some IRB experts view risks in secondary-data only research. These differences in risk perception are consequential. Requests for waiver of informed consent often hinge on perceptions about what constitutes “minimal risk.” These determinations affect whether research proceeds, is exempt under Common Rule standards, or if a waiver of informed consent requirements is appropriate. One participant candidly noted that IRBs at different institutions might evaluate risk differently:

*“Database only studies with access to PII and involve sensitive information may constitute greater than minimal risk in some conservative institutions while other institutions would only look at the gathering of data as research, which would constitute minimal risk.”*

These differences were apparent to our participants after reviewing anonymous peer comments. For example:

*“I’m surprised at how many respondents had no concerns.”*

*“I am not sure why the response is not acceptable to an IRB. It seemed clear to me.”*

*“Very interesting on how much variation there is across the respondents.”*

While our template IRB application and template responses received strong support from respondents, participants disagreed often on the content and level of detail of the documents, and even the purview of IRB review. We attempted to address conflicting feedback as best possible—often by adding more content—but this backfired for some participants who found the revised language “excessive” or “overstated.” Accordingly, we are skeptical about whether universal IRB template language can address the substantial variability in risk assessment and content preference we observed in the IRB community. As one participant noted, *“I am never completely satisfied.”*

Nevertheless, we are confident that this language will facilitate communications with IRBs about research using privacy-enhancing RL software. Improving IRB understanding of cutting-edge technology, like MiNDFIRL, may improve approval rates and speed.

### Research ethics contribution to privacy-by-design

The development of privacy-enhancing technology for secondary data research depends on effective communication and collaboration between the research ethics experts and technology developers. Privacy is a complex issue that requires a holistic approach that is best addressed by designing the information system with the privacy considerations in mind from the beginning, a principle known as privacy-by-design.^16^^,^^39^

Privacy expert participation in this design process is important yet often neglected. We note that the NGT and Delphi methods are participant driven. Selecting these methods helped faithfully integrate these important perspectives into the final template application and responses. Perhaps more importantly, these research ethics perspectives helped guide MiNDFIRL’s final design. These engagements ultimately supported designing the interface to support the “minimum necessary” privacy principle that allows for appropriate access to needed data. Beyond privacy-by-design, our engagement with research ethics experts in the design process facilitated an ethics-by-design product, MiNDFIRL.

### Limitations

While we aimed to have representation of research ethics professionals from a variety of settings, there were no participants from for-profit IRBs or outside of the United States, who may provide additional perspectives. Given that vigorous discussions about RL have unfolded at the international level,^16^^,^^40–43^ perspectives on MiNDFIRL from the international research ethics community may warrant future research.

## Conclusion

Communication between researchers and IRB professionals is not always easy in highly technical database research. There are gaps in understanding both technical issues and ethical concerns. Moreover, as our study shows, risk perceptions can be variable between different IRB professionals. When these gaps are not addressed, research is often delayed unnecessarily, at the expense of scarce resources and frustration. Effective communication can bypass these harms, and our findings can help bridge the knowledge gaps between researchers and IRB professionals and facilitate more efficient dialogue and approval of database research involving RL.

To the best of our knowledge, this research is the first case study engaging the research ethics community as part of the participatory design of software to facilitate transparency and communication. Our study contributes to the limited research identifying the perspectives of an important group of professionals on privacy-enhancing software. The NGT approach allowed the participants to divulge information in a robust way both independently for idea generation and collectively for consensus building. We used these results to draft the initial prototype IRB template form and draft PI responses. Then the Delphi technique that followed allowed us to solicit feedback from experts and improve on the documents, which we disseminated with the MiNDFIRL software. The developed template language can help researchers better understand what information (benefits, risks, etc) is desired by IRB members and how to effectively convey it. We posit that software disseminated with such companion documents will enhance transparency and accountability in the RL process and that MiNDFIRL will constitute a positive step in that direction.

## Supplementary Material

ooae010_Supplementary_Data

## Data Availability

Some of data underlying this article cannot be shared publicly to protect the privacy of individuals that participated in the study. Other data will be shared on reasonable request to the corresponding author.
