# Supplementary material for: Case study on communicating with research ethics committees about minimizing risk through software: an application for record linkage in secondary data analysis
Source: JAMIA Open. 2024 Feb 29;7(1):ooae010. doi: 10.1093/jamiaopen/ooae010 (PMC10903982; doi:10.1093/jamiaopen/ooae010)
Supplement: ooae010_Supplementary_Data [file ooae010_supplementary_data.zip › irb_app_template_mindfirl.pdf]

IRB APPLICATION TEMPLATE FOR STUDIES DOING SECONDARY DATA ANALYSIS INVOLVING RECORD LINKAGE

PI Instructions: Below is a sample IRB application for secondary data studies with template language relating to the MINDFIRL record linkage software. If you plan to use MINDFIRL for record linkage in your study protocol, you might find this language useful in communicating with the IRB about the software, which was developed with input from the IRB community. **The suggested language below only relates to the use of MINDFIRL and does not include information that is specific to your protocol.** You should add details about your study, applicable laws, and information that might be required by your institution's policies and procedures. MINDFIRL is also a configurable software that includes settings that enhance protections if desired. If you plan to use specific software configurations in your study, you should modify the suggested language below accordingly. We recommend that you include copies of all agreements between project personnel and the data custodians (i.e., data use agreements) in your IRB application. We note that the IRB will likely review your protocol under the assumption that the research team will access all PII capable of disclosure through MINDFIRL.. Additionally, **we recommend that you talk with your institution's IT department about MINFIRL and ensure that your installation and use of the MINDFIRL software is compliant with your institution's IT standards.**

1. **IRB Protocol Title: ___________**

2. **Investigator and Contact Person**

   a. **Name of Principal Investigator:**
      Degree(s)/Title:
      Dept/Division:
      Phone:
      Email:
      Mailing Address:

   b. **Name of Contact Person:**
      Title:
      Email:
      Phone:

3. **Protocol Personnel:**

   a. **Organizational Personnel**

**Name, Degree, and Department**	**Role**	**Access to PII for linkage**	**Access to Coded Data (i.e., analysis)**	**Access to the Key linking PII to coded data**	**Financial Interest?**	**Protocol Responsibilities and Qualifications**
Name:<br>Degree:<br>Department		☐ Yes<br>☐ No	☐ Yes<br>☐ No	☐ Yes<br>☐ No	☐ Yes<br>☐ No	
Name:<br>Degree:<br>Department		☐ Yes<br>☐ No	☐ Yes<br>☐ No	☐ Yes<br>☐ No	☐ Yes<br>☐ No	

   b. **Non- Organizational Personnel**

Name, Degree, and Department	Role	Access to PII for linkage	Access to Coded Data (i.e., analysis)	Access to the Key linking PII to coded data	Financial Interest?	Protocol Responsibilities and Qualifications	From institution with or without own IRB
Name:<br>Degree:<br>Department		☐ Yes<br>☐ No	☐ Yes<br>☐ No	☐ Yes<br>☐ No	☐ Yes<br>☐ No		☐ Has own IRB but requests that [this organization] serve as IRB of record.<br>☐ Does not have own IRB and will rely on [this organization's] IRB
Name:<br>Degree:<br>Department		☐ Yes<br>☐ No	☐ Yes<br>☐ No	☐ Yes<br>☐ No	☐ Yes<br>☐ No		☐ Has own IRB but requests that [this organization] serve as IRB of record.<br>☐ Does not have own IRB and will rely on [this organization's] IRB

c. **Are any of the investigators listed above students using this research for their thesis or dissertations?**

☐ Yes
☐ No

Student Name	Thesis/Dissertation Title

4. Funding

Is this protocol funded?

☐ Yes
☐ No

If yes, attach a copy of completed application or request for funding sent to sponsor, and complete a-d.

a. Title of Grant, Contract, or Agreement:
b. PI of Grant, Contract, or Agreement:
c. Office of Sponsored Programs Assigned Number:
d. Sponsor/Funding Source:
   ☐ Government Agency or Agencies:
   ☐ NIH
   ☐ Private non-profit:
   ☐ Industry, investigator-initiated:

5. Locations Involved

a. Indicate all organizations that will provide data for the conduct of this protocol:

   ☐ X
   ☐ X

b. Describe the organizations that will provide data for the conduct of this protocol identified in 5(a).

c. Indicate all organizations that will provide space or facilities for the conduct of this protocol:

- ☐ X
- ☐ X

d. Describe the organizations that will provide space or facilities for the conduct of this protocol identified in 5(c)

6. Multi-site Studies

a. Is this a multi-site study with [this organization] as the lead investigator?

- ☐ Yes
- ☐ No

b. Is this a multi-site study with [this organization] as a coordinating site?

c. If Yes to a or b, describe the management of information obtained from various sites that might be relevant to the protection of participants. Include, at a minimum, how the following items are managed:

- ☐ IRB approvals from other sites
- ☐ Unanticipated problems involving risks to participants or others (For e.g., if there is an unanticipated problem involving risks to participants, which site is responsible for reporting it?
- ☐ Interim results
- ☐ Protocol modifications

7. What are the research questions or hypotheses to be studied?

8. Please describe briefly how this study will contribute to existing knowledge in the field.

9. Purpose of the research – in non-technical, lay language

a. Summarize the purpose and objectives of this protocol in one short paragraph

b. Describe how outcomes will be measured for this protocol

10. Background – in non-technical, lay language

Summarize in 2-3 paragraphs past research findings leading to the design of this protocol. Include any relevant past or current research by the PI.

11. Data Element Use, Management, and Protection

a. Will the PI or others obtain, review, or make other use of participant information that is protected under any federal, state, or international law (e.g., HIPAA, FERPA, CIPSEA, GDPR)?

- ☐ Yes
- ☐ No

b. Indicate which of the entities would provide legally protected information for this protocol, maintain legally protected information as it was collected for this protocol, and/or store legally protected information after it has been collected for this protocol.

- ☐ X
- ☐ X
- ☐ X

c. Indicate which of the listed identifiers will be accessed, associated and/or linked with the protected health information (PHI) used for this protocol.

- ☐ Names
- ☐ Geographic subdivisions smaller than a state
- ☐ Elements of dates (except year) related to an individual
- ☐ Telephone numbers
- ☐ Fax numbers
- ☐ Email addresses
- ☐ Social security numbers
- ☐ Medical record numbers
- ☐ Health plan beneficiary numbers
- ☐ Account numbers
- ☐ Certificate/license numbers
- ☐ Vehicle identifiers and serial numbers
- ☐ Device identifiers and serial numbers
- ☐ Biometric identifiers
- ☐ Web universal resource locators (URLs)
- ☐ Internet protocol address numbers
- ☐ Full-face photographic images
- ☐ Any other unique identifying number – Describe: _________

d. Justify your need to link participants' data by using identifying information.

e. Describe how and where the data will be stored and how the location where data are stored will be secured in your absence. If applicable, state specifically where any IRB-approved or participant-signed consent documents will be kept for 3 years after the study ends.

f. Describe the network location or hard drive on which the documents will be saved/stored.
- ☐ Is the network location describe above secured or encrypted?
  - ☐ Yes
  - ☐ No

[**PI Instructions**: The following three questions (g, h, i) are important for you to be able to answer and share with the IRB. Find the most appropriate questions in your IRB application to share this information]

g. Which IT department is managing the server that has MINDFIRL installed and will store the PII?

h. Has the IT department reviewed and approved the use of MINDFIRL?

i. Who maintains the MINDFIRL access logs? Who has access to the logs? Who will review the logs? How often?

j. Describe how electronic data will be secured during transmission.

k. Will any study data be stored on an external/removal storage device?

- ☐ Yes
- ☐ No
  - If yes, where will the external/removable storage device be kept?
  - Who will have access to the external/removable storage device?
  - Will the external/removable storage device be encrypted?

l. Explain when the identifiable data will be destroyed or the identifiers will be removed.

m. How will the confidential data be destroyed?

12. Participants

a. How many participants will provide data for this protocol?
List number from each site
- ☐ X
- ☐ X

List total number from all institutions: ___________

b. Describe the characteristics of anticipated or planned participants.

c. From what population(s) will participants be derived?

d. Describe the inclusion/exclusion criteria.

e. Indicate which, if any, of the special populations listed below be involved in the protocol.

- ☐ Pregnant women
- ☐ Fetuses
- ☐ Neonates
- ☐ Prisoners
- ☐ Minors (<18 years old)
- ☐ Employees or students at institution where research will be conducted
- ☐ Persons who are temporarily decisionally impaired
- ☐ Persons who are permanently decisionally impaired
- ☐ Illiterate, limited, or no English language proficiency

For each box checked, explain why the group is included and the additional protections to protect the rights and welfare of these participants who are vulnerable to coercion.

f. Describe the recruitment process that will be used to seek the data of potential participants.

g. If you will use recruitment materials to reach the owners of data for potential participants, attach a copy of each item.

13. **Protocol procedures, methods, and duration – in non-technical, lay language**

   a. Describe the procedures for all aspects of your protocol. Tell us what you are doing.

PI Response:

"The data used for this study is subject to the following laws: [**PI Instructions**: list applicable state or federal laws]. Accordingly, this research will follow the following policies and procedures to ensure compliance with the law: [**PI Instructions**: list any organizational or study-specific policies and procedures]. [PI Instructions: if any research data is subject to a data use agreement or other contractual restrictions, mention those restrictions here and include the agreement as an attachment.]

[**PI Instructions**: You should state the full protocol for your study here. The template language below only relates to conducting record linkage using MINDFIRL. You can incorporate this language in your description of the protocol as appropriate.]

We will use the MINDFIRL software to link data from different databases, namely [**PI Instructions:** list databases]. MINDFIRL will be used to facilitate data linkage of PII while controlling researcher access to PII and coded sensitive data to minimize identity exposure and unnecessary privacy loss. See Section 17 below for specific steps to enhance privacy and confidentiality and Attachments A and B for details relating to MINDFIRL.

[**PI Instructions**: if this study will use the Privacy Loss Limit function of the MINDFIRL software to place an upper limit on discretionary PII unmasking (i.e., to further limit privacy risk), you should indicate it here and include the following language: "We will use tools within the MINDFIRL software to restrict disclosure of certain PII to researchers. For additional details regarding these protections see section 17 below."] The Privacy Loss Tracking Report indicates how specific researchers used the MINDFIRL software to access PII for record linkage. However, no PII is included in the summary report. This information will provide transparency in access to PII as well as quantify the actual privacy risk associated with the linkage process.

[**PI Instructions:** We recommend that a designated person on the project review the Privacy Loss Tracking Report at least annually. Please state here, who on the project team will have the responsibility of reviewing the Privacy Loss Tracking Report, and how often it will be reviewed.] If required by the IRB, the Privacy Loss Tracking Report can be provided to the IRB (e.g., continuation review)."] An example of a MINDFIRL Privacy Loss Tracking Report can be found in the last page of **Attachment A** ."

   b. What is the probable length of time required for the entire protocol (recruitment, acquisition of the data, analysis, and study closure)?

14. **Benefits**

   a. Describe the potential benefits, if any, to participants or to society from this project.

15. Risks

a. From the list below, please select ALL of the potential risks that are involved with this study.

- ☐ Physical risks
- ☐ Psychological risks
- ☐ Social/Economic risks
- ☐ Loss of confidentiality (e.g. potential for breach of confidentiality such that non-authorized personnel gain access to private information such as educational or medical records.

b. Describe the known risks for participants as a result of participation in the research, i.e. persons whose data will be used. If the study has no greater than minimal risk (e.g., those risks ordinarily encountered in daily life or during the performance of routine physical or psychological examinations or tests), describe why this is so.

PI Response:

"[**PI Instructions**: Carefully consider whether there are any unique concerns or considerations with this research that might increase the risks associated with this study above minimal risk. Survey responses from a panel of IRB professionals suggest that the inclusion of the following types of information might make a database-only research greater than minimal risk:

- Contagious disease information (e.g., STDs, HIV status)
- Information associated with social stigma (e.g., illegal activity, criminal history, substance abuse)
- Mental or behavioral health information
- Genetic information
- Sexual activity

Based on the nature of the data used you should consider mentioning the following categories of participant risks: Social/Economic risks; Possible invasion of privacy of subject or subject's family; Psychological risks; Physical risks]

[**PI Instructions**: the following template language is intended for database-only research ***without*** any unique concerns or considerations that would increase the risks associated with this study above minimal risk.] Because this is a database only study, there will be no contact with study participants, and PII data access will only be possible through MINDFIRL, and be restricted to approved personnel only. Loss of confidentiality is the principle risk of this study. The risk of unauthorized re-identification in the linked dataset is expected to be somewhat greater than the risk of unauthorized re-identification of the unlinked datasets primarily because there will be more information in the linked records. Additionally, any data linkage errors increase the risk of inaccurate conclusions related to data subjects. Nevertheless, the nature of this risk is not substantively different from risks experienced in ordinary life, where data is ubiquitous and different datasets are linked for various purposes. As such, this is a minimal risk study as it poses no greater risks than those ordinarily encountered in daily life. The use of the MINDFIRL software will further reduce risk to the minimum necessary to conduct reliable record linkage. Despite the use of the MINDFIRL software, which aims to reduce unnecessary disclosures for purposes of record linkage, all database studies have an inherent risk of unexpected disclosures due to a potential breach of the system."

c. Do you foresee that participants might need additional medical or psychological resources as a result of your research procedures?

- ☐ Yes
- ☐ No

If yes, describe the provisions that have been made to make these resources available.

d. Do the benefits or knowledge to be gained outweigh the risks to participants?

- ☐ Yes
- ☐ No

If no, provide justification for performing the research.

16. **Precautions/Minimization of Risks**

   a. Describe precautions that will be taken to avoid or minimize risks to protect the welfare of participants and the mechanisms for monitoring to detect risks.

PI Response:

The main risk in this database study is the loss of privacy and confidentiality. We discuss procedures to protect privacy and confidentiality in the following section – section 17.

   b. Describe how the research team will handle an adverse or unexpected outcome that could be potentially harmful.

17. **Procedures to Protect Privacy and Confidentiality**

   a. Describe how you will protect the privacy interests of participants. Include how you will ensure unnecessary privacy loss or identity exposure to authorized personnel.

PI Response:

"We will be using the new MINDFIRL software to reduce the risks of unauthorized PII disclosures (both internally and externally) during record linkage. The MINDFIRL software was developed with input from IRB, legal, and ethics experts and patients living with chronic diseases, who by virtue of their conditions have a high likelihood of personal records stored by various health organizations. MINDFIRL minimizes risk during the record linkage process by disclosing only the necessary information needed to make linkage decisions, tracking who accessed what part of the data, and managing the data in a coded manner such that the PII and sensitive data is kept separate.

MINDFIRL masks all PII, but helps researchers link records by 1) using picture clues to help researchers understand if two masked values are the same or how they are different, 2) allowing researchers to selectively unmask data values if they believe it will help them accurately link records, 3) track and log all access to PII, and 4) limit access to certain amount of PII (i.e., no access is 0% and total access is 100%, and a limit can be specified in between for total access) or details of the PII (e.g., only X digits of the SSN).

The software has a clickable interface that allows users to open identifying information on an 'as needed' basis in order to come to a firm linkage decision. As such, team members conducting the record linkage process will make decisions on whether records from multiple databases belong to the same person by accessing identifying information incrementally, ultimately leading to the disclosure of fewer subject identifiers to research personnel. **Attachment A** includes a demonstration of MINDFIRL and the incremental access of PII.

Researchers authorized to conduct record linkage will only be able to access the PII and unmask PII as needed for record linkage through the MINDFIRL software. Their access will be authenticated following institutional policies. See Template Question 3 above for a list of study personnel permitted to access PII. The MINDFIRL software maintains all PII in a coded manner. MINDFIRL restricts researcher access to the encryption "key" capable of connecting PII with the coded sensitive data. The key is securely maintained inside MINDFIRL, and only the following personnel, [**PI Instructions**: list the personnel who will have access to the code that can connect the PII to the sensitive data; this may be an IT person not on the project.], will have access to the code for emergency recovery.

In addition, MINDFIRL records and logs all unmasked PII to help understand (1) who has accessed what PII, and (2) how much total PII was accessed (i.e., the level of risk). MINDFIRL uses an algorithm to assess the privacy risk associated with unmasked PII based on how likely it is to identify the real person. Generally, this algorithm assigns a higher privacy risk score to unique values within a data set and lower privacy loss score to common values. For example, the risk of unmasking Bob is much lower than the risk of unmasking Hye-Chung. [**PI Instructions**: if this study will use the Privacy Loss Limit function of the MINDFIRL software to place an upper limit on discretionary PII unmasking (i.e., to further limit privacy risk), you should indicate it here and include the following language: "We will be configuring the MINDFIRL software to use the Privacy Loss Limit function in order to enforce that overall the project will have no more than [**PI Instructions:** specify the total project limit that will be

used] privacy loss. In addition, we setup MINDFIRL such that [**PI Instructions:** you should describe the specific limits on disclosure here (e.g., only four digits on the SSN). Given the guidelines in MINDFIRL, we reviewed the quality of the data and estimate this will be sufficient to have good record linkage while providing enhanced privacy"].

Our use of the MINDFIRL software for record linkages indicates our commitment to limit privacy loss. Because this is a database only study, we will further minimize risk through appropriate data management protocols and practices such as authenticated access (e.g., password protected) to the data, and ensuring that the data remain behind the institution's firewall on a secure server at all times."

b. Describe how you will store research data to maintain confidentiality, including how access is limited. If data will be stored electronically anywhere than a server maintained by [this organization], identify the organization, department, and all computer systems use to store protocol-related data.

PI Response:

[**PI Instructions:** if relevant, indicate how any study data will be given or transmitted to the study team]. Once we receive the data, we will be using a server maintained by [**PI Instructions**: specify who maintains the study server to store and analyze PII for record linkage purposes.] Linked data will be maintained in a coded manner using MINDFIRL and access will be restricted using authentication. Similarly, MINDFIRL will restrict access to PII and the encryption "key" capable of connecting the PII with the coded data using authentication.

Only the specified subset of personnel listed in this application involved in linkage will have access to the PII through MINDFIRL. See Template Question 3 above for a list of study personnel who has access to PII and who has access to the encryption key. For additional information on MINDFIRL see **Attachment A**.

[**PI Instructions:** describe the specific technical controls (i.e., security) that will be used to protect confidential data, e.g., password-protection, two-factor authentication, firewall, data retention/destruction]"

Additionally, MINDFIRL includes software tools to support transparency, accountability, and to discourage unnecessary PII unmasking.

c. Describe the administrative controls that will be in play for the carrying out of this research protocol, including training and user rules.

PI Response:

"All study personnel engaging in the record linkage process have undergone [this organization's] required human subjects research training which equips them with the knowledge of administrative controls and requirements and the consequences that non-adherence to these requirements poses.

The MINDFIRL software allows for specific tracking of the identifying information seen by each linkage staff member, so unauthorized disclosures can be more easily decreased, tracked and investigated [**PI Instructions:** if appropriate, direct the IRB to question 13 above for the monitoring plan for the Privacy Loss Tracking Report]. Thus, in using MINDFIRL for record linkage, the research team will adhere to responsible and accountable data governance and data use. If a report indicates that a research personnel is disclosing more PII than should be expected during record linkage (e.g., compared to peers), the PI will attempt to determine why and propose a corrective plan (e.g., training) when necessary. Unauthorized breaches of PII will be promptly reported to the IRB.

[**PI Instructions:** List specific administrative controls at your organization]. In addition, all study personnel engaged in record linkage will be trained in using MINDFIRL. See Attachment B for details on the MINDFIRL training. "

18. Storage for future use and sharing data beyond this project

a. Will the linked data from this protocol be stored for future use?

   ☐ Yes
   ☐ No

   If **yes**, answer the following questions:

   i. Indicate whether the linked data will be PII, coded, or de-identified.

   ii. Describe where the data will be stored.

   iii. Describe how the data will be stored.

   iv. What IRB is responsible for overseeing the stored data?

b. Will any data from this protocol be given to any person, including the subject, or any group, including coordinating centers and sponsors?

   If **yes**:

   ☐ Who will receive the data?

   ☐ What data will be shared?

   ☐ Is the shared data PII, coded, or de-identified?
