# Supplementary material for: Case study on communicating with research ethics committees about minimizing risk through software: an application for record linkage in secondary data analysis
Source: JAMIA Open. 2024 Feb 29;7(1):ooae010. doi: 10.1093/jamiaopen/ooae010 (PMC10903982; doi:10.1093/jamiaopen/ooae010)
Supplement: ooae010_Supplementary_Data [file ooae010_supplementary_data.zip › MINDFIRL_training_Delphi_ELSI.pdf]

MINDFIRL Static Tutorial

For the dynamic tutorial with the full experience, visit:
http://newtutorial.herokuapp.com/?mode=4

MINDFIRL Training

- Users of the MINDFIRL software will have to complete an online training module to familiarize themselves with the interface and the record linkage procedure using MINDFIRL. This training utilizes two publicly available datasets with realistic pairs of records to identify the same people among the generated data pairs. The training includes an overview of record linkage, MINDFIRL features and functions and how it facilitates record linkage while ensuring privacy protection.
- Additionally, the training requires users get a hands-on experience on using MINDFIRL to conduct record linkage through practice problems (i.e., they are asked to make decisions about whether record pairs belong to the same or to different person. The tutorial can be repeated as needed, until the future user is confident about using the data of their own interest.
- The software is currently in prototype, so the training is not finalized. However, the training will be similar to the existing tutorial for the prototype MINDFIRL. This tutorial is available at:

  http://ppirl-tutorial.herokuapp.com/introduction

- The slides used in the tutorial are attached for convenience.

Introduction to Record Linkage

Same or Different People?

Data entry collects information about people. Your job in this study is to:
1) Look at pairs of rows of data about people
2) Decide whether or not the pair refers to the same person.

Pair	ID	First name	Last name	DoB(M/D/Y)	Sex	Race
1	8000002767	JUDE	WILLIAM	09/09/1906	M	W
	8000003567	JUDE	WILLIAM JR	09/09/1960	M	B
2	0000006947	BRYANT	MADELINE	05/02/1962	F	W
	0000006947	MADELINE	BRYANT	05/02/1962	F	W
3	9000018540	SALLY	BYRD	07/04/1960	F	W
	6000008928	JOHN	BYRD	04/07/1960	M	

Common Issues with Data about People

Watch out for common issues

Data are expressed differently

- Nick Names (Elizabeth & Beth)

Data change over time

- Women get married and change their last name

Data are not unique attributes

- John Smith (there are different people that have the same name)
- Twins & Family members have similar identifying information such as DOB & last name
- Same names in Families with different suffix (Jr and Sr)

Data are sometimes missing

- SSN are often missing

Data have errors

- Inserting/deleting extra characters
- Typing in the wrong character
- Transposing two characters
- First name and last name are mixed up

Missing Values

Data are sometimes missing.

Pair	ID	First name	Last name	DoB(M/D/Y)	Sex	Race
7	0000018335	PATSY	CALLAHAN	11/13/1948	F	B
		PATSY	CALLAHAN		F	B

Added or Deletions Characters

Insertion (or deletion) of characters are common typing errors

Pair	ID	First name	Last name	DoB(M/D/Y)	Sex	Race
1	8000001276	JAYDEN	TIPTON	09/09/1960	M	W
	8000002768	JADEN	TIPTON	09/09/1960	M	W

Different Characters

Mistyping can lead to certain characters replacing others

Pair	ID	First name	Last name	DoB(M/D/Y)	Sex	Race
3	9000018540	SAL	BYRD	04/07/1960	F	W
	9000018870	SAL	BIRD	04/09/1960	F	W

Switched Characters

Two characters can be interchanged by mistake

Pair	ID	First name	Last name	DoB(M/D/Y)	Sex	Race
11	1719582520	ROGRES	HYLEMON	07/15/1924	M	W
	1719852520	ROGERS	HYLEMON	07/15/1942	M	W

Column Swaps

Due to mix up, sometimes whole values are swapped as well

Date Swaps

Pair	ID	First name	Last name	DoB(M/D/Y)	Sex	Race
9	0000020502	SAMANTHA	MORGAN	02/11/1958	F	W
	0000020502	SAMANTHA	MORGAN	11/02/1958	F	W

Name Swaps

Pair	ID	First name	Last name	DoB(M/D/Y)	Sex	Race
5	0000006947	BRYANT	MADELINE	09/22/1926	F	W
	0000006947	MADELINE	BRYANT	09/22/1926	F	W

Different

Sometimes the values are very different

Pair	ID	First name	Last name	DoB(M/D/Y)	Sex	Race
13	6556368585	WILL	GREENE	07/03/1950	M	B
	1092091430	DAVE	GREENE	07/03/1950	M	W

Common and Rare Names

It can be helpful to consider how common or unique a person's name is.

For example, consider how common these names might be in the United States:

Very Common First Names	Uncommon First Names
Michael	Brooklynn
Matthew	Jamarion
Mary	Jaxson
Ashley	Araceli

Very Common Family Names	Uncommon Family Names
Smith	Febland
Jones	Pober
Jackson	Southwark
Williams	Raynott

Name Frequency

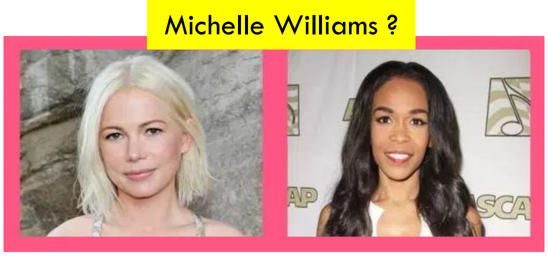

It would not be surprising for two people to have the same **common name**, but it might be unlikely for two people to have the same **rare names**.

How to use Name Frequencies

Pair	ID	First name	Last name	DoB(M/D/Y)	Sex	Race
1	8000002767	JOHN	SMITH	09/09/1906	M	W
	✖					
	8000003567	JOHN	SMITH	09/09/1906	M	W
2	0000006847	DEQUAN	WAMBOLDT	05/02/1962	F	W
	✖					
	0000006947	DEQUAN	WAMBOLDT	05/02/1962	F	W

For **pair 1**, despite the ID being pretty similar, the chances that both the records refer to the same person are **pretty low** since **John Smith** is a **common name**.

For **pair 2**, which has pretty similar conditions except for the name frequency, the chances are indeed much **higher** since **Dequan Wamboldt** is a **unique name**!

Decision Making

Deciding if two rows are the same person is not a simple "yes" or "no" decision. You have to **think in terms of chance**. Let's take an example:

Pair	ID	First name	Last name	DoB(M/D/Y)	Sex	Race
1	8000001276	JAYDEN	TIPTON	09/09/1960	M	W
	8000002768	JADEN	TIPTON	09/09/1960	M	W

We don't know for sure if these two rows refer to the same person. You should ask yourself:

> "**What are the chances** that two rows refer to the same person when the ID and the first name have small differences and all other info same?"

The chances are pretty high that this is the same person, but you still cannot be 100%.

How to Give Your Answer

Next, we explain how you will give you answer for each pair of people.

Pair	ID	First name	Last name	DoB(M/D/Y)	Sex	Race	Choice Panel
1	8000002767	JUDE	WILLIAM	09/09/1906	M	W	H M L L M H
	8000003567	JUDE	WILLIAM JR	09/09/1960	M	B	Different Same
2	0000006947	BRYANT	MADELINE	05/02/1962	F	W	H M L L M H
	0000006947	MADELINE	BRYANT	05/02/1962	F	W	Different Same
3	9000018540	SALLY	BYRD	07/04/1960	F	W	H M L L M H
	6000008928	JOHN	BYRD	04/07/1960	M		Different Same

The Answer Buttons

Different | **Same**

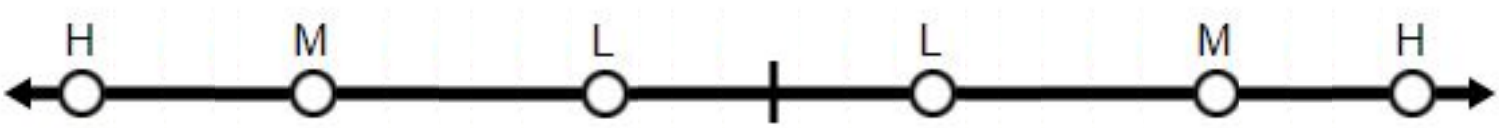

Highly Likely Different	Moderately Likely Different	Less Likely Different	Less Likely Same	Moderately Likely Same	Highly Likely Same

If you think the rows are the **same person**, click one of the choices on the **right side**. Pick one of L, M, H depending on your confidence level.

If you think the rows are for **different people**, click one of the choices on the **left side**. Pick one of L, M, H depending on your confidence level.

Ready to Give it a Try?
Let's do Some Practice Problems

The next step will be to do some practice problems.

Ready to continue?

Click on button below to confirm you have gone through all the slides, and click the next button to move onto the practice problems.

Practice Problems I:
Nature of ID numbers

In most cases,

- When **IDs are identical**, they usually refer to the **same person**
  - Even if there are some differences in identifying information such as:
    - Nick names
    - Women often change their last names due to marriage
- When **IDs are different**, the usually refer to **different people**
  - Even if the IDs might seem similar
  - Father and son have same first name and last name. Sometimes suffixes (Jr/Sr/III) are available to help along with birthdates that should be roughly 30 years apart.

Pair	ID	FFreq	First Name	Last Name	LFreq	DoB(M/D/Y)	Sex	Race	Choice Panel
1	9320952205	•••	EMMA	BRIGGS	∞	12/29/1987	F	W	H M L L M H
				DIFF					
	9320952205	•••	EMMA	DEYTON	∞	12/29/1987	F	W	Different Same
2	1299747019	1	ERNESTO	PEDROZA SR	1	04/19/1964	M	O	H M L L M H
	DIFF			×		DIFF			
	6456839076	1	ERNESTO	PEDROZA JR	1	07/23/1997	M	O	Different Same

Easy? Where we need human judgement

- Using IDs to make decisions will work in many situations.
- But there are particularly difficult situations that do not always follow the basic principle:
  - **Identical IDs can refer to different people (e.g., twins)**
  - **Different IDs can refer to the same person (e.g., duplicate records)**

In the following slides, we will learn about these difficult situations.

Practice Problems II:
Data Errors in IDs

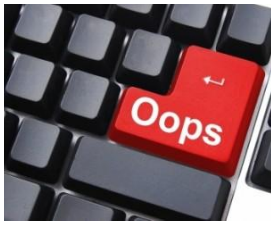

Typos and common data entry errors in ID numbers can results in similar IDs referring to the same person

- **Adding** extra characters or **deleting** characters
- Typing in the **wrong character**
- **Switching** two characters

Pair	ID	FFreq	First Name	Last Name	LFreq	DoB(M/D/Y)	Sex	Race	Choice Panel
	1742682819	•••	SARA	BOONE	∞	05/21/1988	F	W	H M L L M H
1	+			+					
	1742668281	•••	SARA	STYLES-BOONE	1	05/21/1988	F	W	Different Same
	1466919280	•••	TAYLOR	DELLINGER	•••	06/12/1988	F	W	H M L L M H
2	⇄					✕			
	1466991280	•••	TAYLOR	DELLINGER	•••	12/06/1988	F	W	Different Same

Practice Problems III:
Nature of Twin Data Records

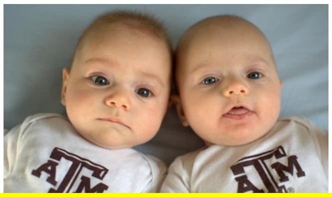

Can you tell the twins apart?
Remember, **twins are two different people!**

- Twins and family members who share similar identifying information pose problems.
- **Twins can have the same ID** due to mix ups, and they also share very similar information (same last name, birthdates, similar first name).
- Twins and family members often have similar IDs because the **IDs are issued together**.

Pair	ID	FFreq	First Name	Last Name	LFreq	DoB(M/D/Y)	Sex	Race	Choice Panel
	1777743279	•••	ALEXANDER	BROST	25	05/04/1994	M	W	H M L L M H
1	✕		++				DIFF		
	1777743278	25	ALEXANDRA	BROST	25	05/04/1994	F	W	Different Same
	2224902624	•••	SAMUEL	MASTERS	25	01/27/1945	M	W	H M L L M H
2	✕		+						
	2224902524	•••	SAM	MASTERS	25	01/27/1945	M	W	Different Same

Practice Problems IV: Duplicates IDs

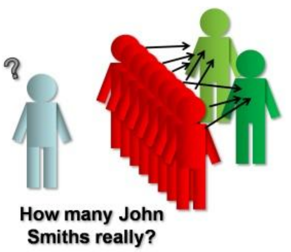

- Sometimes, databases have multiple entries for the same person because the system does not realize that the person already exists in it.
- Then, the database can have **two IDs that are totally different that refer to the same person**. These records are often called duplicates.
- But pairs that are legitimately two different people can have similar identifying information, and these should **not be confused with duplicates IDs.** Examples include:
  - Twins
  - Father and son have same first name and last name. Sometimes suffixes (Jr/Sr/III) are available to help along with birthdates that should be roughly 30 years apart.
  - Other family members

Pair	ID	FFreq	First Name	Last Name	LFreq	DoB(M/D/Y)	Sex	Race	Choice Panel
1	7755370155	•••	KEITH	ROBINSON	∞	07/31/1958	M	W	H M L L M H
	DIFF		DIFF				DIFF		
	5811750761	•••	KELLY	ROBINSON	∞	07/31/1958	F	W	Different Same
2	5678412359	1	RUFORD	SWANSON	25	05/16/1961	M	B	H M L L M H
	DIFF					⇄			
	4897541253	1	RUFORD	SWANSON	25	05/16/1916	M	B	Different Same

Practice Problems V: Missing IDs

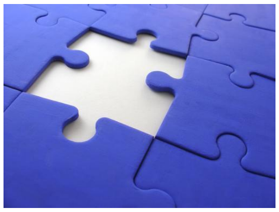

Sometimes IDs can be **missing**. You will then have to use the other information to help you make the decision.

For example, how likely do you think it is to have two different people:

- With the exact same first name and last names, except with the names swapped?
- Born on the same exact date?
- Living in a county together?

Pair	ID	FFreq	First Name	Last Name	LFreq	DoB(M/D/Y)	Sex	Race	Choice Panel
1	?	•••	GEORGE	THOMAS	∞	12/23/1972	M	W	H M L L M H
						DIFF			
	1201397313	∞	THOMAS	GEORGE	•••	11/26/1935	M	W	Different Same
2	1856554310	1	GAILYA	OMONDI	1	09/29/1978	F	W	H M L L M H
	?	1	OMONDI	GAILYA	1	09/29/1978	F	W	Different Same

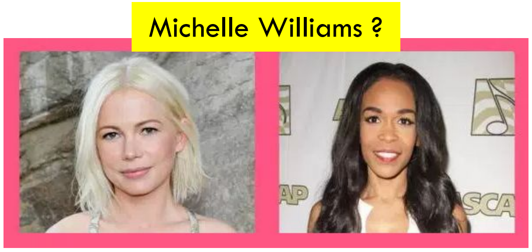

Practice Problems VI: Frequency of names

Another key information to consider when making decisions is **how common the names** are and the **race distribution**.

For example, how likely do you think it is to have two different people with:

- The **exact same first name and last name** when it is a **common** name (John Smith)
- The **exact same first name and last name** when it is a **rare** name (Dequan Wamboldt)

Pair	ID	FFreq	First Name	Last Name	LFreq	DoB(M/D/Y)	Sex	Race	Choice Panel
1	2325174462	1	MENDEZ	NICOLAS	1	08/04/1976	M	H	H M L L M H
	?	25	NICOLAS	MENDEZ	25	08/04/1976	M	H	Different **Same**
2	?	•••	CASEY	FOX	∞	07/18/1989	F	B	H M L L M H
							DIFF	DIFF	
	6049349211	•••	CASEY	FOX	∞	07/18/1989	M	W	**Different** Same

VISUAL MASKING FOR PRIVACY

Wait! There is more ...
What about Privacy ?

Due to privacy concerns, **you will not see all the identifying information** in the table, but you should have sufficient information to make decisions.

The upcoming pages will give you an understanding of how the data is disclosed on a need-to-know basis.

Checkmarks: Identical Values ✓

Identical values are shown as checkmarks.

Pair	ID	FFreq	First name	Last name	LFreq	DoB(M/D/Y)	Sex	Race
	1234567891	•••	BRIAN	TIPTON	∞	09/09/1960	F	W
1	✗		+					
	1234561291	2-5	BRIANNA	TIPTON	∞	09/09/1960	F	W

Pair	ID	FFreq	First name	Last name	LFreq	DoB(M/D/Y)	Sex	Race
	******@@**	•••	*****	✓	∞	✓	✓	✓
1	✗		+					
	******&&**	2-5	*****&&	✓	∞	✓	✓	✓

Stars Used in Similar IDs

When two items are similar, **stars** (they look like this ***) are used for characters that are the **same**.

@@@ and &&& show the characters that are **different**.

Pair	ID	FFreq	First name	Last name	LFreq	DoB(M/D/Y)	Sex	Race
1	1234567891	•••	BRIAN	TIPTON	∞	09/09/1960	F	W
	✖		+					
	1234561291	2-5	BRIANNA	TIPTON	∞	09/09/1960	F	W

Pair	ID	FFreq	First name	Last name	LFreq	DoB(M/D/Y)	Sex	Race
1	******@@**	•••	*****	✓	∞	✓	✓	✓
	✖		+					
	******&&**	2-5	*****&&	✓	∞	✓	✓	✓

*** When the Matched Value is Missing ()

When one of the values in a pair is missing, the other value is not useful.

Thus, the matched value with missing is represented by ***

Pair	ID	FFreq	First name	Last name	LFreq	DoB(M/D/Y)	Sex	Race
7	0000018335	•••	PATSY	CALLAHAN	•••	11/13/1948	F	B
	?	•••	PATSY	CALLAHAN	•••	?	F	B

Pair	ID	FFreq	First name	Last name	LFreq	DoB(M/D/Y)	Sex	Race
7	**********	•••	✓	✓	•••	**/**/****	✓	✓
	?	•••	✓	✓	•••	?	✓	✓

Different Items

@@@@@@@@@@
DIFF
&&&&&&&&&&

When two items are very different, they are shown as @@@ and &&&

Pair	ID	FFreq	First name	Last name	LFreq	DoB(M/D/Y)	Sex	Race
13	6556368585	1	WILL	GREENE	•••	07/03/1950	M	B
	DIFF		DIFF					DIFF
	1092091430	1	DAVE	GREENE	•••	07/03/1950	M	W

Pair	ID	FFreq	First name	Last name	LFreq	DoB(M/D/Y)	Sex	Race
13	@@@@@@@@@@	1	@@@@	✓	•••	✓	✓	@
	DIFF		DIFF					DIFF
	&&&&&&&&&&	1	&&&&	✓	•••	✓	✓	&

Swaps

When columns have swapped values, the swapped parts are shown by &&& and @@@

Pair	ID	FFreq	First name	Last name	LFreq	DoB(M/D/Y)	Sex	Race
5	0000006947	1	BRYANT	MADELINE	1	02/05/1962	F	W
	0000006947	2-5	MADELINE	BRYANT	•••	05/02/1962	F	W

Pair	ID	FFreq	First name	Last name	LFreq	DoB(M/D/Y)	Sex	Race
5	✓	1	&&&&&&	@@@@@@@@	1	@@/&&/****	✓	✓
	✓	2-5	@@@@@@@@	&&&&&&	•••	&&/@@/****	✓	✓

Understand the Symbols?

Take a moment to look at the next slide carefully and understand what the symbols and icons mean for different values.

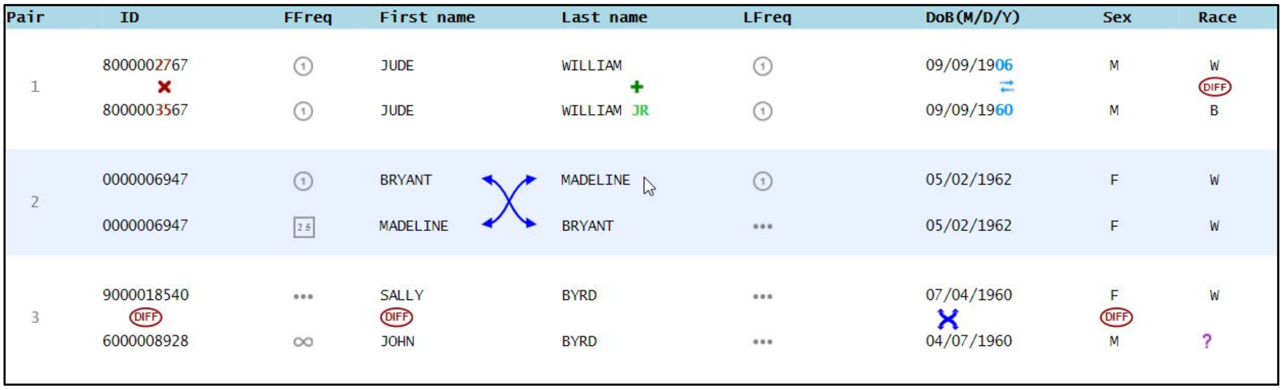

Pair	ID	FFreq	First name	Last name	LFreq	DoB(M/D/Y)	Sex	Race
1	8000002767	①	JUDE	WILLIAM	①	09/09/1906	M	W
	✖			+		⇄		DIFF
	8000003567	①	JUDE	WILLIAM JR	①	09/09/1960	M	B
2	0000006947	①	BRYANT	MADELINE	①	05/02/1962	F	W
	0000006947	2-5	MADELINE	BRYANT	•••	05/02/1962	F	W
3	9000018540	•••	SALLY	BYRD	•••	07/04/1960	F	W
	DIFF		DIFF			✖	DIFF	
	6000008928	∞	JOHN	BYRD	•••	04/07/1960	M	?

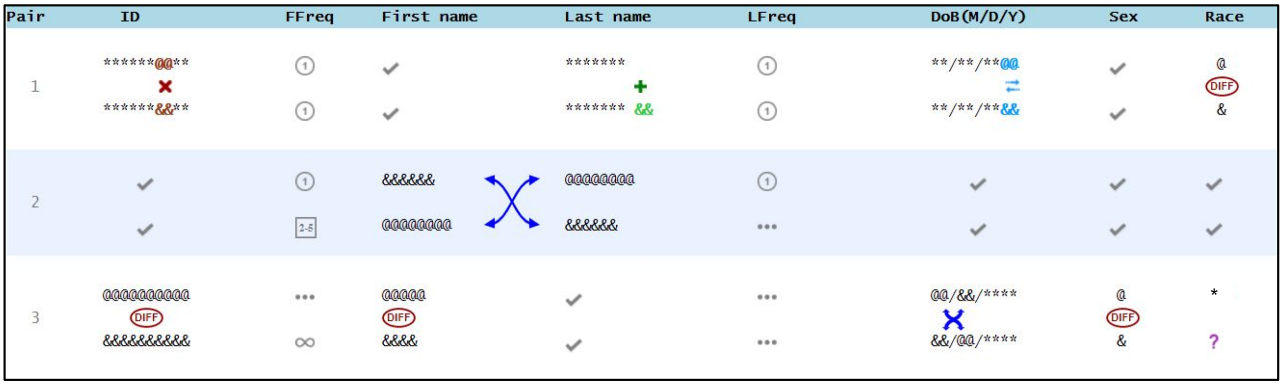

Pair	ID	FFreq	First name	Last name	LFreq	DoB(M/D/Y)	Sex	Race
1	******@@**	①	✓	*******	①	**/**/**@@	✓	@
	✖			+		⇄		DIFF
	******&&**	①	✓	******* &&	①	**/**/**&&	✓	&
2	✓	①	&&&&&&	@@@@@@@@	①	✓	✓	✓
	✓	2-5	@@@@@@@@	&&&&&&	•••	✓	✓	✓
3	@@@@@@@@@@	•••	@@@@@	✓	•••	@@/&&/****	@	*
	DIFF		DIFF			✖	DIFF	
	&&&&&&&&&&	∞	&&&&	✓	•••	&&/@@/****	&	?

Practice

Pair	ID	FFreq	First Name		Last Name	LFreq	DoB(M/D/Y)	Sex	Race	Choice Panel
1	✓	•••	✓		@@@@@@	∞	✓	✓	✓	H M L L M H
					DIFF					
	✓	•••	✓		&&&&&&	∞	✓	✓	✓	Different Same
2	@@@@@@@@@@	25	✓		********@*	1	@@/@@/@@@@	✓	✓	H M L L M H
	DIFF				✗		DIFF			
	&&&&&&&&&&	25	✓		********&*	1	&&/&&/&&&&	✓	✓	Different Same
3	*********@	•••	*******@*		✓	25	✓	@	✓	H M L L M H
	✗		++					DIFF		
	*********&	25	********&		✓	25	✓	&	✓	Different Same
4	**********	1	@@@@@@	⤫	&&&&&&	1	✓	✓	✓	H M L L M H
	?	1	&&&&&&		@@@@@@	1	✓	✓	✓	Different Same

Answers

We have opened all the records for you to review.

Pair	ID	FFreq	First Name		Last Name	LFreq	DoB(M/D/Y)	Sex	Race	Choice Panel
1	9320952205	•••	EMMA		BRIGGS	∞	12/29/1987	F	W	H M L L M H (H on Same selected)
					DIFF					
	9320952205	•••	EMMA		DEYTON	∞	12/29/1987	F	W	Different **Same**
2	1299747019	25	ERNESTO		PEDROZA SR	1	04/19/1964	M	O	H M L L M H (H on Different selected)
	DIFF				✗		DIFF			
	6456839076	25	ERNESTO		PEDROZA JR	1	07/23/1997	M	O	**Different** Same
3	1777743279	•••	ALEXANDER		BROST	25	05/04/1994	M	W	H M L L M H (H on Different selected)
	✗		++					DIFF		
	1777743278	25	ALEXANDRA		BROST	25	05/04/1994	F	W	**Different** Same
4	1856554310	1	GAILYA	⤫	OMONDI	1	09/29/1978	F	W	H M L L M H (H on Same selected)
	?	1	OMONDI		GAILYA	1	09/29/1978	F	W	Different **Same**

In Pair 1, the only differences is the last name, which can often be due to marriage for females.
In Pair 2, both the ID and DOB is very different. In additions, even the last name has differences.
In Pair 3, the same last name (which seems to be a rare name) and DOB, with similar ID and first name with sex being different is good evidence for twins.
In Pair 4, everything is the same except ID is missing and unique name seems to be swapped providing evidence for data entry error in name.

Clickable Interface

Interactive On-Demand Interface

That was hard, wasn't it?

Sometimes, data masking can hide data that might be essential for record linkage. What if **you could open up the masked data as you need to see more**?

Over the next few pages, we will walk you through an interactive on-demand interface for record linkage.

Video on clickable interface

http://newtutorial.herokuapp.com/static/images/tutorial/clickable/demo.mp4

Before cell click

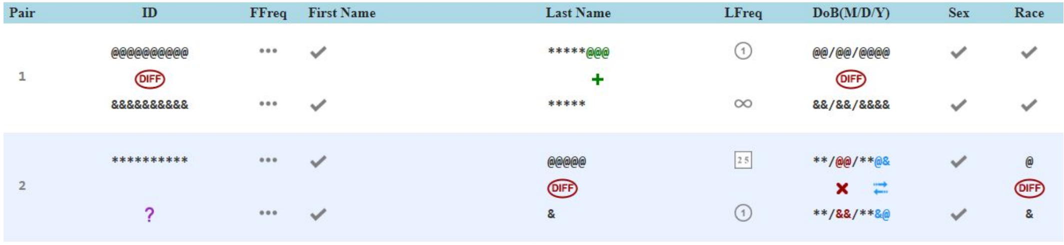

After cell click

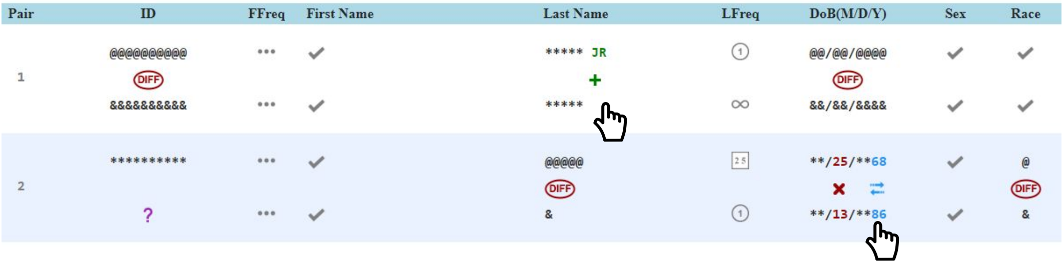

More Information with One Click

Did you pay attention to how cells were clicked open?

For cells that are **completely identical** or **completely different**, all the contents will be **fully opened in one click**.

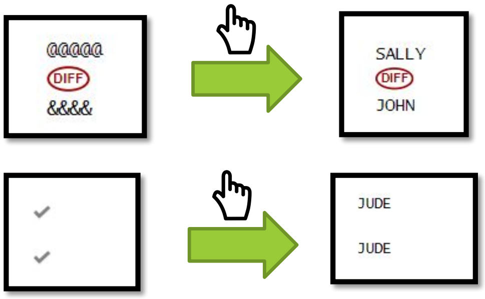

More Information with Two Clicks

For cells that are partly different, one click will only show details for the different parts. A second click will show the full information.

That means **partially different cells can be clicked twice to open them fully**
Remember, you might not need to see it all.
In the first example, you probably only need to see the JR and not William

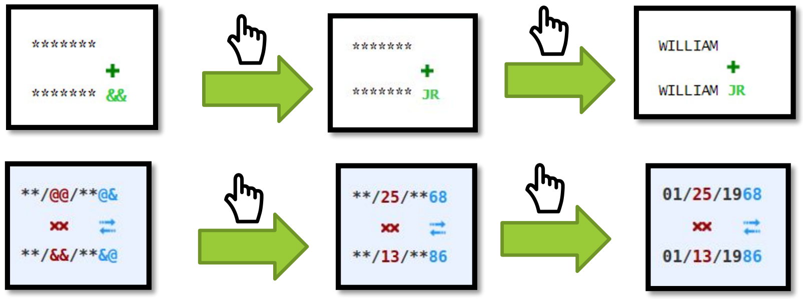

What to open?

Remember, you should open the relevant cells you need to make the correct linkage decision.

Do not open cells unless you think it will help you make better decisions but at the same time, if you need information to make good linkage decisions, go ahead and open it!

You will be given the most credit for correctly answering the most questions while opening up only the relevant cells.

Let's look at an example next.

What to open video

http://newtutorial.herokuapp.com/static/images/tutorial/clickable/decision_making.mp4

What to open? (If you saw the video skip this slide)

First let's take a look into the problem.

Everything except the ID, DoB and Last Name are identical.

The mentioned fields are similar though not identical.

Pair	ID	FFreq	First Name	Last Name	LFreq	DoB(M/D/Y)	Sex	Race	Choice Panel
	?	①	@@@@@@@@@	✓	∞	**/**/**@*	✓	✓	H M L L M H
1			DIFF			✗			←O—O—O+O—O—O→
	**********	∞	&&&&&&	✓	∞	**/**/**&*	✓	✓	Different Same

Next, let us look at the DoB on the next page

What to open? (If you saw the video skip this slide)

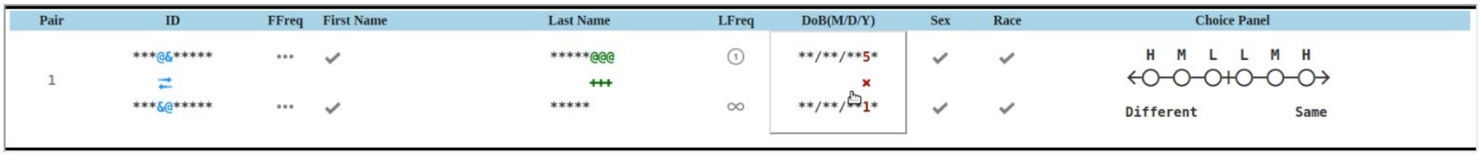

Date of birth (DoB) has a difference of almost 40 years!

Next, let's look at the differences in the Last Name field on the next page

What to open? (If you saw the video skip this slide)

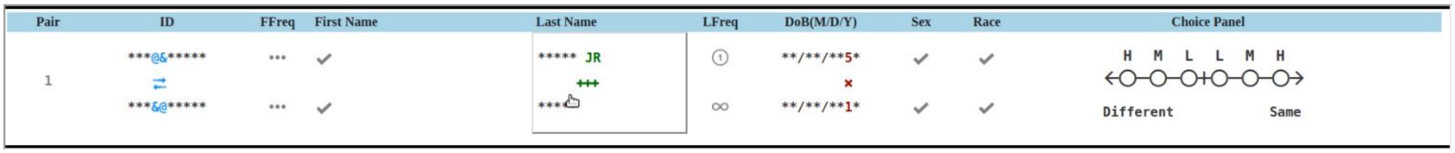

A 'junior' in the Last Name? Perhaps father and son?

Let's take a look at the gender to confirm.

What to open? (If you saw the video skip this slide)

Pair	ID	FFreq	First Name	Last Name	LFreq	DoB(M/D/Y)	Sex	Race	Choice Panel
	@&**	•••	✓	***** JR	①	**/**/**5*	M	✓	H M L L M H
1	⇄			+++		×			
	&@**	•••	✓	*****	∞	**/**/**1*	M	✓	Different Same

The gender is male, indeed! This supports our hypothesis about it being father and son.

What to open? (If you saw the video skip this slide)

Pair	ID	FFreq	First Name	Last Name	LFreq	DoB(M/D/Y)	Sex	Race	Choice Panel
	@&**	•••	✓	***** JR	①	**/**/**5*	M	✓	H M L L M H
1	⇄			+++		×			
	&@**	•••	✓	*****	∞	**/**/**1*	M	✓	Different Same

We don't need to open any more information to make our decision. These two are likely father and son, so they are different people.

What NOT to open?

If you don't need to see the details to make a decision, DO NOT open it.

You may not need to open anything to make a decision, as in the example below.
Opening up the swapped name does not give you more information to make a decision.

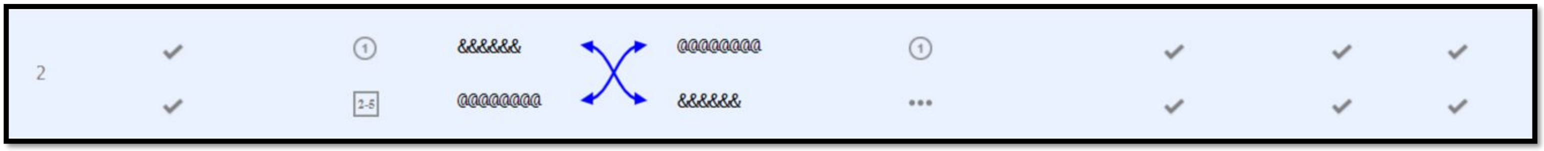

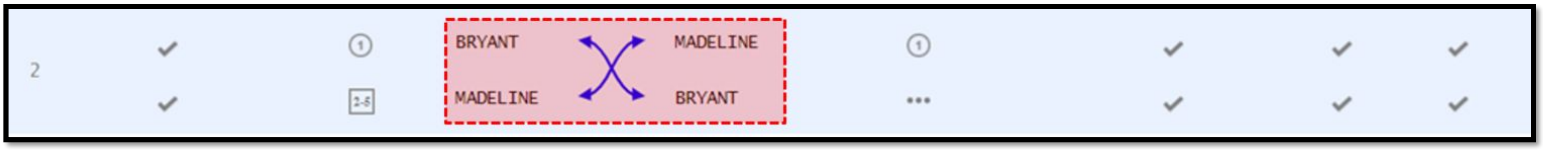

When you might want to open identical values?

Sometimes you might need to see the full items even when you know the values are the same:

- Often females change their last name but males do not

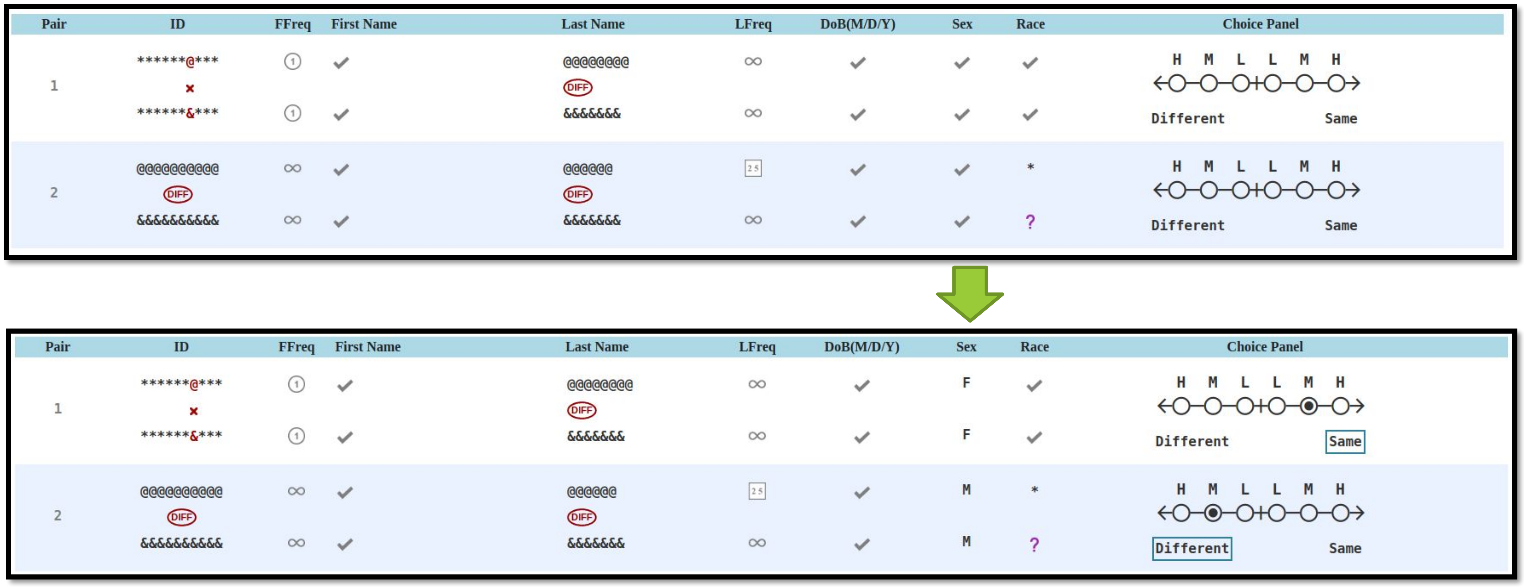

Practice

Now try to make record linkage decisions as best you can.

Be smart and try to only open cells you need to see to make a good decision.

Pair	ID	FFreq	First Name	Last Name	LFreq	DoB(M/D/Y)	Sex	Race	Choice Panel
1	@@@@@@@@@@<br>DIFF<br>&&&&&&&&&&	1<br>1	✓<br>✓	@@@@@<br>DIFF<br>&&&&&&	•••<br>25	✓<br>✓	✓<br>✓	✓<br>✓	H M L L M H<br>Different Same
2	✓<br>✓	•••<br>•••	✓<br>✓	*****<br>+<br>*****&&&	•••<br>•••	**/**/**@&<br>⇄<br>**/**/**&@	✓<br>✓	✓<br>✓	H M L L M H<br>Different Same

Answers

Good job!
We have opened all the records for you to review.
Please click the next button when you are done.

Pair	ID	FFreq	First Name	Last Name	LFreq	DoB(M/D/Y)	Sex	Race	Choice Panel
1	1001300066<br>DIFF<br>1001068224	1<br>1	AMBROSIA<br>AMBROSIA	JONES<br>DIFF<br>CUTTER	•••<br>25	07/18/1986<br>07/18/1986	F<br>F	B<br>B	H M L L M H<br>Different Same
2	1000563341<br>1000563341	•••<br>•••	SEBASTIAN<br>SEBASTIAN	SMITH<br>+<br>SMITHSON	•••<br>•••	10/02/1908<br>⇄<br>10/02/1980	M<br>M	W<br>W	H M L L M H<br>Different Same

Privacy Meter

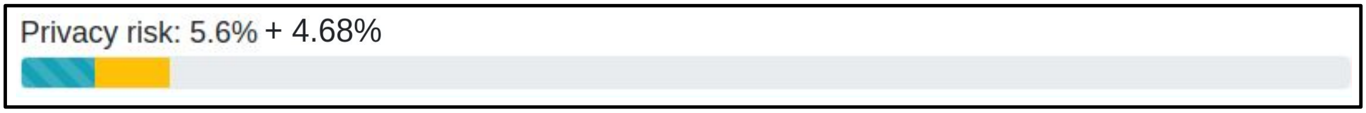

The meter will help you monitor how much you have opened up, and how much you have left.

The blue bar indicates **how much you have opened so far**.

When you mouse over cells you want to open, the orange bar indicates how much the click would "cost".

- If you do not click, it goes away
- If you click, it turns blue

Privacy Meter with Limit

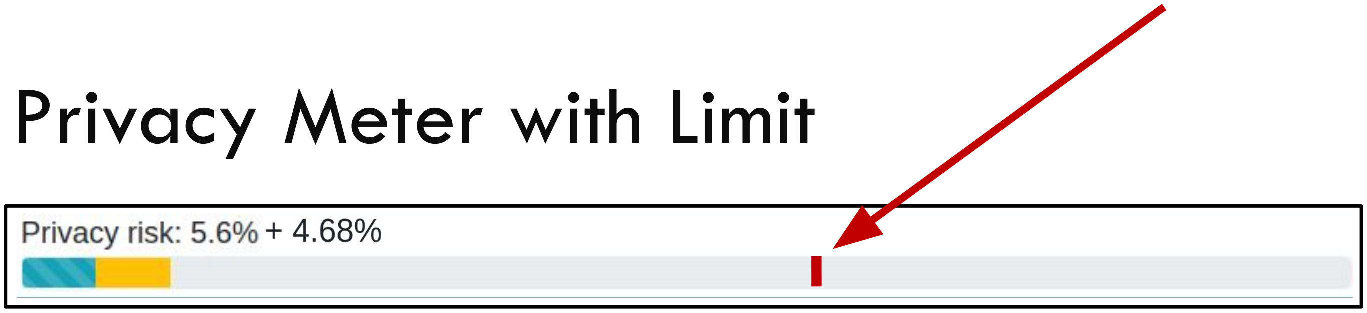

100% (the full meter) is when all cells are fully open

What is the **solid red line on the meter**?

- This is the **maximum budget you have to spend (open up cells)**
- You will not be able to open anything else after you reach the solid red line.
- If you reach the bar, then for the rest of the questions, you'll have to make the best choice you can without opening anything else.
- Be careful to only open cells that you need to make your decision.

How should you budget?

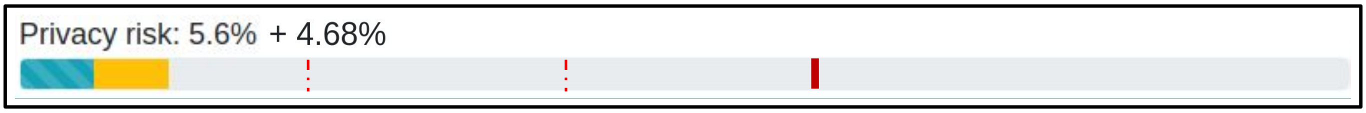

Let’s say you have 18 questions, 6 a page. You have a total up to the solid red line to spend on answering all 18 questions over 3 pages

So, try not to use all of it on the first page.

Instead, try to spend roughly 1/3 on each page.

Practice 3

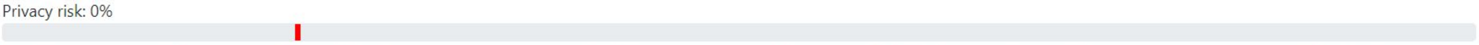

Pair	ID	FFreq	First Name	Last Name	LFreq	DoB(M/D/Y)	Sex	Race	Choice Panel
1	✓<br>✓	25<br>25	*********<br>*********	@@@@@<br>DIFF<br>&&&&	∞<br>25	✓<br>✓	✓<br>✓	✓<br>✓	H M L L M H<br>Different Same
2	@@@@@@@@@@<br>DIFF<br>&&&&&&&&&&	25<br>25	✓<br>✓	****<br>+<br>****&&&	•••<br>25	@@/@@/@@@@<br>DIFF<br>&&/&&/&&&&	✓<br>✓	✓<br>✓	H M L L M H<br>Different Same
3	✓<br>✓	•••<br>•••	***@@@<br>✗<br>***&&&	✓<br>✓	•••<br>•••	✓<br>✓	@<br>DIFF<br>&	@<br>DIFF<br>&	H M L L M H<br>Different Same
4	*******@&*<br>⇄<br>*******&@*	①<br>①	✓<br>✓	✓<br>✓	①<br>①	@@/&&/****<br>✗<br>&&/@@/****	✓<br>✓	✓<br>✓	H M L L M H<br>Different Same

Answers

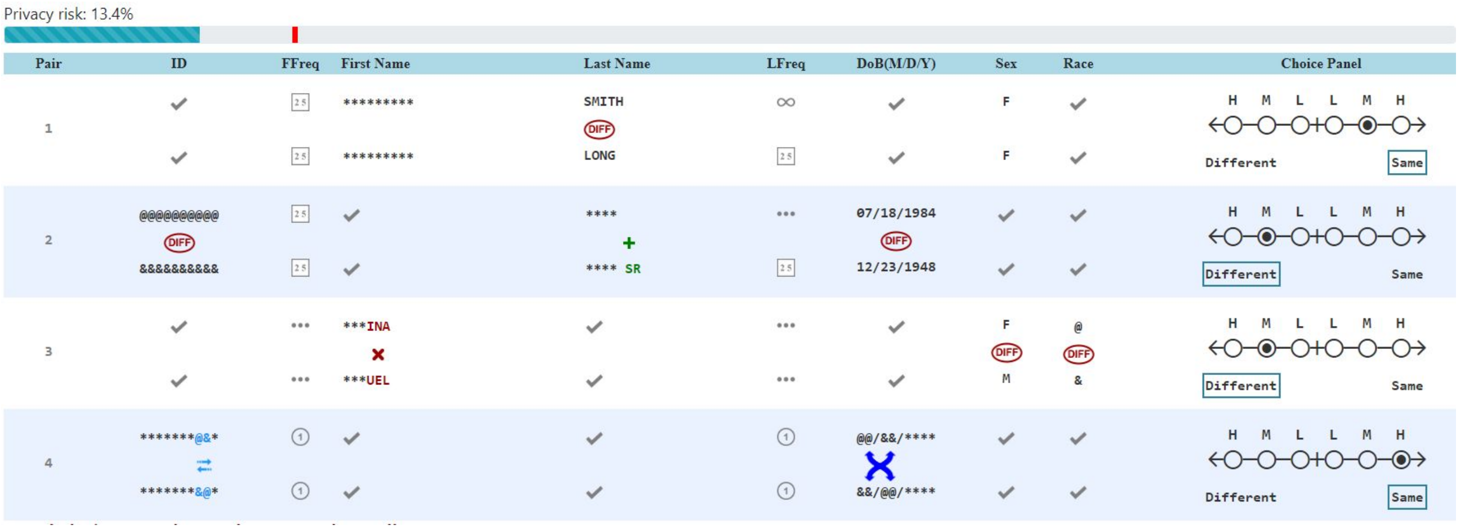

Other links

- To experience the clickable interface, please visit: https://ppirl2.herokuapp.com/
- To experience the same tutorial in a dynamic fashion which gives the full experience, please visit http://newtutorial.herokuapp.com/?mode=4
