# Supplementary material for: Case study on communicating with research ethics committees about minimizing risk through software: an application for record linkage in secondary data analysis
Source: JAMIA Open. 2024 Feb 29;7(1):ooae010. doi: 10.1093/jamiaopen/ooae010 (PMC10903982; doi:10.1093/jamiaopen/ooae010)

What is MINDFIRL?

- User interface for record linkage that masks identifiers, but allows users to reveal additional information about identifiers selectively on an 'as needed' basis

- While the masked data is fully de-identified, graphical cues help users determine how similar two records are and the nature of any differences

Current Status of Record Linkage

ID	First name	Last name	DoB(M/D/Y)	Sex	Race
8000002767	JUDE	WILLIAM	09/09/1906	M	W
8000003567	JUDE	WILLIAM JR	09/09/1960	M	B

- Many research protocols require access to fully identifiable information to link data (see above)
- Full disclosure without markup
- Highest privacy risk

MINDFIRL: Symbols used in the Software

Highlight discrepancies

- ? Empty fields
- ✖ Different characters
- ✚ Extra characters
- ⇄ Transposed values
- ⤨ Name or Date Swaps
- DIFF Major field differences

Name frequency meta-data

- ① Unique
- 2 5 Rare
- ••• Common
- ∞ Highly common

MINDFIRL:
Masked disclosure with markup

- Clickable interface: allows partial disclosure of necessary information for more accurate record linkage

Pair	ID	FFreq	First name	Last name	LFreq	DoB(M/D/Y)	Sex	Race
1	******@@**<br>✖<br>******&&**	①<br>①	✓<br>✓	*******<br>✚<br>******* &&	①<br>①	**/**/**@@<br>⇄<br>**/**/**&&	✓<br>✓	@<br>DIFF<br>&
2	✓<br>✓	①<br>2-5	&&&&&&<br>⤨<br>@@@@@@@@	@@@@@@@@<br>&&&&&&	①<br>•••	✓<br>✓	✓<br>✓	✓<br>✓
3	@@@@@@@@@@<br>DIFF<br>&&&&&&&&&&	•••<br>∞	@@@@@<br>DIFF<br>&&&&	✓<br>✓	•••<br>•••	@@/&&/****<br>✕<br>&&/@@/****	@<br>DIFF<br>&	@<br>?

How does it work?

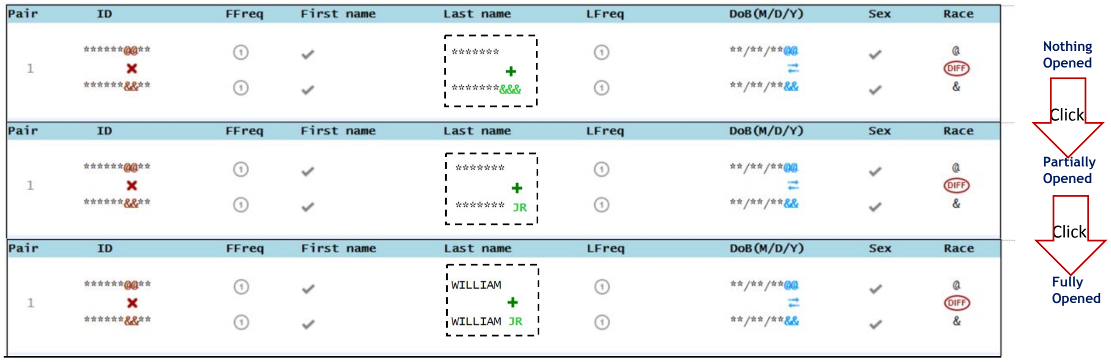

How does it work?

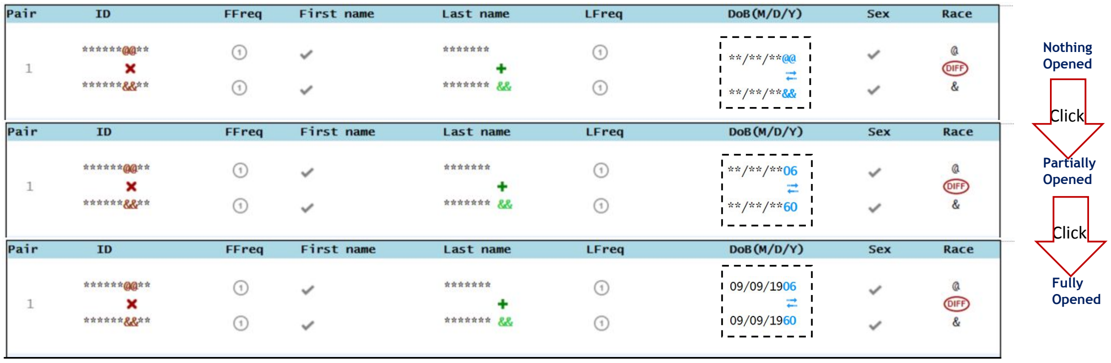

Supplement: ooae010_Supplementary_Data [file ooae010_supplementary_data.zip › IRB_Delphi_slides to accompany IRB template.pdf]
